# Comparative accuracy of pleural fluid unstimulated interferon-gamma and adenosine deaminase for diagnosing pleural tuberculosis: A systematic review and meta-analysis

**DOI:** 10.1371/journal.pone.0253525

**Published:** 2021-06-24

**Authors:** Ashutosh Nath Aggarwal, Ritesh Agarwal, Sahajal Dhooria, Kuruswamy Thurai Prasad, Inderpaul Singh Sehgal, Valliappan Muthu

**Affiliations:** Department of Pulmonary Medicine, Postgraduate Institute of Medical Education and Research, Chandigarh, India; Federal University of Santa Catarina, BRAZIL

## Abstract

**Objective:**

We compared diagnostic accuracy of pleural fluid adenosine deaminase (ADA) and interferon-gamma (IFN-γ) in diagnosing tuberculous pleural effusion (TPE) through systematic review and comparative meta-analysis.

**Methods:**

We queried PubMed and Embase databases to identify studies providing paired data for sensitivity and specificity of both pleural fluid ADA and IFN-γ for diagnosing TPE. We used hierarchical summary receiver operating characteristic (HSROC) plots and HSROC meta-regression to model individual and comparative diagnostic performance of the two tests.

**Results:**

We retrieved 376 citations and included 45 datasets from 44 publications (4974 patients) in our review. Summary estimates for sensitivity and specificity for ADA were 0.88 (95% CI 0.85–0.91) and 0.91 (95% CI 0.89–0.92), while for IFN-γ they were 0.91 (95% CI 0.89–0.94) and 0.96 (95% CI 0.94–0.97), respectively. HSROC plots showed consistently greater diagnostic accuracy for IFN-γ over ADA across the entire range of observations. HSROC meta-regression using test-type as covariate yielded a relative diagnostic odds ratio of 2.22 (95% CI 1.68–2.94) in favour of IFN-γ, along with better summary sensitivity and specificity figures. No prespecified subgroup variable significantly influenced the summary diagnostic accuracy estimates.

**Conclusion:**

Pleural fluid IFN-γ estimation has better diagnostic accuracy than ADA estimation for diagnosis of TPE.

## Introduction

Tuberculosis (TB) remains an important etiology of exudative pleural effusions, especially in regions with high TB burden [1]. However, it is often difficult to establish a definite diagnosis of tuberculous pleural effusion (TPE). Owing to the paucibacillary nature of this disease, microbiological confirmation of TPE from pleural fluid specimens (smear, culture, or nucleic acid amplification tests) is suboptimal [1]. Pleural biopsy can demonstrate mycobacteria, or typical caseating granulomatous inflammation, in a higher proportion of patients. However, it is an invasive procedure and not routinely performed, especially in resource-constrained settings.

Pleural fluid adenosine deaminase (ADA) is a widely used biomarker for TPE. ADA, a purine degrading enzyme mostly found in T-lymphocytes, has good accuracy for diagnosing TPE. In a meta-analysis of 174 studies, we reported a summary sensitivity and specificity of 0.92 and 0.90 respectively [2]. Although the assay is simple, inexpensive, and widely available, an optimal threshold for pleural fluid ADA is still not clear. In areas with high burden of TB, the presence of elevated pleural fluid ADA (commonly >40 IU/L) in patients with lymphocytic exudative effusions is usually considered sufficient to initiate empiric anti-tubercular treatment (ATT) [1].

Pleural fluid interferon-gamma (IFN-γ) has also been evaluated as a diagnostic marker for TPE. IFN-γ is a cytokine released from activated CD4+ T-lymphocytes and natural killer cells and has potent anti-mycobacterial activity. We found summary sensitivity and specificity of 0.93 and 0.96 respectively in our recent meta-analysis of 67 studies reporting on use of unstimulated pleural fluid IFN-γ for diagnosing TPE [3]. Compared to ADA assays, IFN-γ testing is complex, considerably more expensive, and hence is not widely employed. Like ADA testing, clinically useful thresholds are also not well-defined for IFN-γ assays [3].

Overall, our summary diagnostic accuracy estimates for unstimulated pleural fluid IFN-γ (notably specificity) appeared superior to those for pleural fluid ADA, as was also suggested in an earlier meta-analysis [2–4]. However, indirect comparison of summary estimates from studies evaluating different patient datasets and heterogenous study designs is likely to yield biased interpretation [5]. A comparative meta-analysis should preferably be restricted to studies applying both diagnostic tests to the same individuals while using a common reference standard [6]. A recent narrative review tabulated such paired information and inferred better diagnostic utility for pleural fluid IFN-γ as compared to ADA [7]. However, available literature was not systematically reviewed, and a formal diagnostic test accuracy meta-analysis was not undertaken. We formally assessed the relative diagnostic accuracy of these two important diagnostic tests for TPE through a systematic review and comparative meta-analysis of studies reporting diagnostic accuracy data for both assays in the same patients.

## Methods

We pre-registered our study protocol with the PROSPERO database (registration number CRD42020222609) and followed the Preferred Reporting Items for Systematic Reviews and Meta-Analyses (PRISMA) guidelines for this review [8, 9]. Prior approval from our Institutional Ethics Committee was not needed since we obtained only summary information from studies already published.

### Search strategy

We searched the PubMed and EMBASE literature databases till November 30, 2020 without any temporal or language restrictions. We used the following free text search terms: (Tuberculosis, Tubercular, Tuberculous, TB, Mycobacterium, Mycobacterial); (Pleura, Pleural, Pleuritis, Pleurisy, Nonrespiratory, Non-respiratory, Extrapulmonary, Extra-pulmonary); (Interferon, Interferon-gamma, Gamma-interferon, IFN-γ); and (Adenosine deaminase, ADA) for this purpose. We also searched bibliographies of the included studies and recent review articles, as well as our personal records, for any additional relevant publications.

### Study selection

After removing duplicate citations, two reviewers (ANA and RA) screened all titles and abstracts identified from the literature search. We excluded publications not primarily reporting on TPE in human subjects, case reports or case series, letters to editor not describing original observations, conference abstracts, review articles, and editorials. The full texts of publications considered potentially eligible by either reviewer were retrieved for further independent assessment by both.

We included a study for data synthesis if it (a) included patients with TPE and at least one additional etiology of exudative pleural effusion, (b) employed a microbiologic (presence of acid-fast bacilli, or positivity for *M*. *tuberculosis* on nucleic acid amplification tests or culture, in pleural fluid, pleural biopsy or another clinical specimen), histopathologic (pleural biopsy demonstrating granulomatous inflammation), and/or clinical (overall clinical, radiological and laboratory features suggestive of TPE, or adequate resolution of effusion after empiric ATT) reference standard for the diagnosis of TPE, (c) conducted ‘both’ index tests in at least 95% of the patients evaluated by either test, and (d) provided numerical data (or information from which such numerical data could be extracted) on both sensitivity and specificity of both index tests for diagnosis or TPE. If the same patient population was used to provide these diagnostic accuracy figures in more than one publication, only the one describing the largest patient dataset was selected. In case of any disagreement, study inclusion was decided by consensus between the two reviewers.

### Data extraction

We extracted the following data from studies finally eligible for inclusion: study design, year of publication, countries where the studies were carried out, inclusion and exclusion criteria, etiology of non-tuberculous pleural effusions, human immunodeficiency virus (HIV) status, the techniques of IFN-γ and ADA assay and their thresholds, blinding in the study, reference standard, the proportion of patients having confirmed diagnosis of TPE using microbiologic or pathologic criteria (referred to hereafter as having ‘definite TB’), number of subjects in each group, and the number of positive and negative assay results for each category of subjects. Wherever the sensitivity/specificity information was reported for more than one diagnostic threshold, we chose the one with the largest sum of specificity and sensitivity. If any publication reported all necessary data separately for two or more distinct patient populations, we considered each as a separate study.

### Statistical analysis

We calculated the sensitivity, specificity, and diagnostic odds ratio (DOR) for both IFN-γ and ADA from each study and computed corresponding 95% confidence intervals (95% CI) using the Clopper-Pearson method [10]. We used a continuity correction of 0.5 in studies with zero cell frequencies before logarithmic or logit transformations.

Neither IFN-γ nor ADA assays have a common threshold value that is widely used as a clinical discriminator. Various investigators have used a wide range of thresholds to define test positivity for both tests. Hence, Rutter and Gatsonis hierarchical model was used to summarize diagnostic accuracy data across the included studies [6, 11]. As a preliminary analysis, we plotted the sensitivity and specificity data from different studies in receiver operating characteristic (ROC) space, and fitted the hierarchical summary receiver operating characteristic (HSROC) model independently for IFN-γ and ADA assays [6, 12]. If these HSROC plots did not cross each other, then the curve positioned nearer to the upper left-hand corner was considered to be consistently more accurate than the other. If the shape parameter of any curve (beta) was close to zero, then that plot was considered symmetrical with no association between test accuracy and test threshold. We compared the diagnostic accuracy of ADA and IFN-γ using a HSROC meta-regression model by assessing the effect of type of test as a covariate on accuracy, threshold and shape parameters of the HSROC model [6]. We used the likelihood ratio chi-squared statistic to identify significant improvement in model fit. We further assessed the symmetry of the HSROC plots for the two tests by allowing the test type to influence variability in test accuracy and test threshold as random effect, but not shape of curve. If the likelihood ratio chi-squared statistic suggested no significant difference between this plot and the one also adjusting for shape parameter, then the shape of plots for the individual tests were considered similar. In this scenario, or if both individual plots were symmetrical, relative diagnostic odds ratio (RDOR) was used as the summary measure of relative test accuracy of the two index tests [13].

The QUADAS-2 (QUality Assessment of Diagnostic Accuracy Studies, version 2) tool was used to describe methodological quality of all included studies [14]. We expressed heterogeneity using the Higgins’ inconsistency index (*I*^2^) and considered it high for *I*^2^ values exceeding 0.75 [15]. Heterogeneity was further explored through a separate subgroup analysis for each test [12]. For this, data was stratified based on prespecified covariates that included study design (prospective or not), national burden of TB (high or not), the prevalence of TB among all the study subjects (below 50% or more), the robustness of reference standard for TPE (composite clinical criteria or definite TB), nature of non-tuberculous pleural effusions (whether transudates included or not), method of diagnostic assay, and blinding in study. World Health Organization guidelines were used to designate countries as high burden [16]. We evaluated publication bias using Deek’s funnel plot. We used GRADE criteria to report the quality of evidence [17].

We used the statistical package Stata (Intercooled Edition 12.0, Stata Corp, Texas, USA) for data analysis. The MetaDAS macro was used to fit HSROC models through the NLMIXED procedure in SAS software (University Edition version 9.4, SAS Institute Inc., North Carolina, USA) [18]. Statistical significance was assessed at p <0.05.

## Results

### Study characteristics

**Fig 1:**
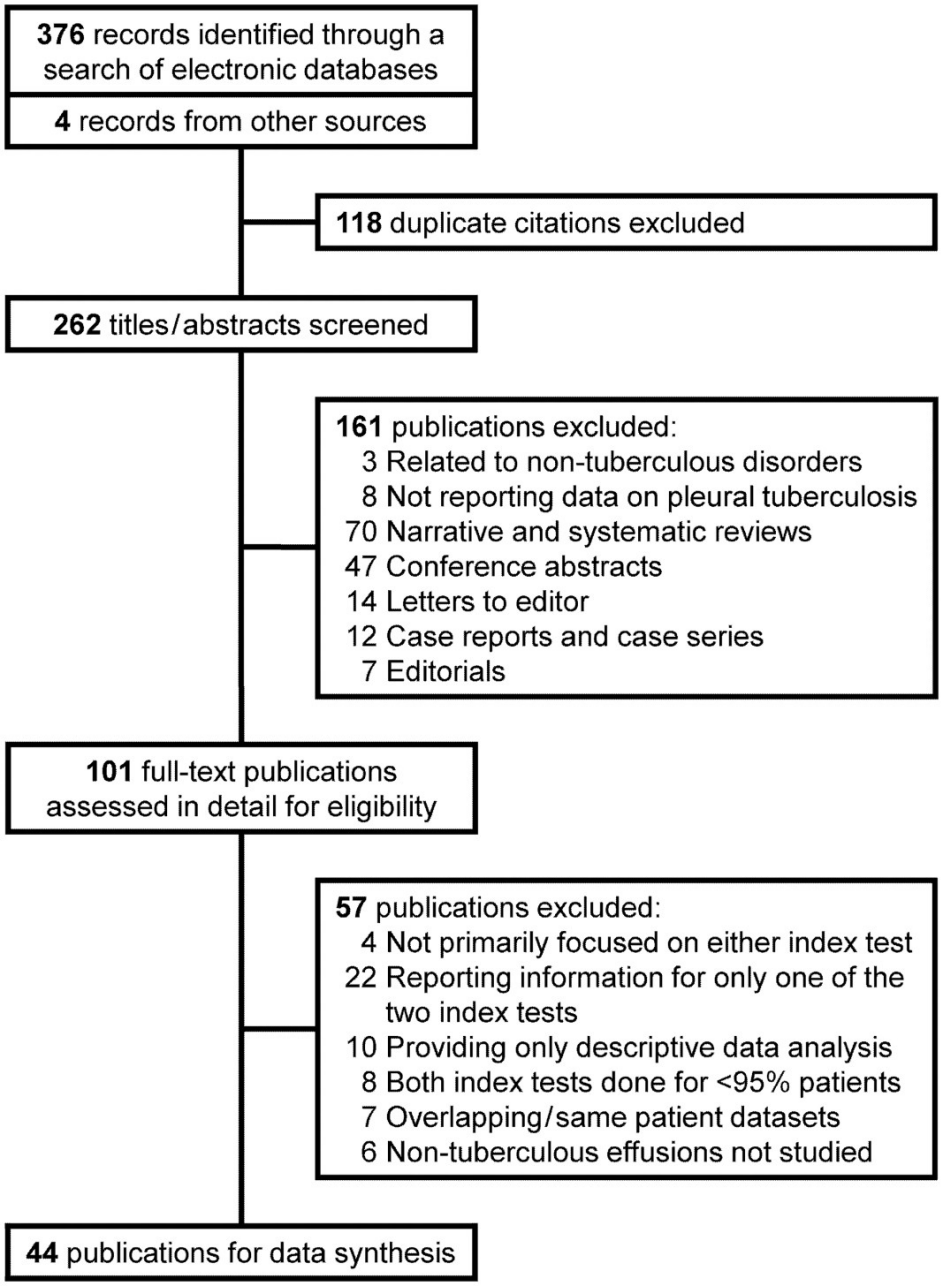
Study selection process.

We identified 376 citations from our electronic database search and added four more from other sources (Fig 1). We evaluated 101 full text publications, and finally included 44 for analysis (Table 1) [19–62]. The reasons for excluding other studies are enumerated in S1 Table. Eight (18.2%) articles were in a foreign language [20, 22–24, 30, 37, 53, 61]. In all, 23 (52.3%) publications were reported from countries having high TB burden [19, 24, 28, 35, 37, 38, 40–45, 48, 49, 53, 55, 56, 58–62]. Blinding was ensured in only four (9.1%) publications [38, 43, 44, 59]. There were no HIV seropositive patients in eight (18.2%) publications [31, 33, 48, 51, 52, 55, 56, 61]. Only two (4.5%) other publications reported the frequency of HIV seropositivity among their study subjects, while the remaining did not provide any information [38, 58]. Most researchers (24, 54.5%) used a definite (microbiologic and/or pathologic) reference criteria for diagnosing TPE [19–21, 23, 26, 29, 31, 33–36, 39, 41, 46, 47, 49–52, 54, 55, 59, 60, 62]. Half of the publications had included patients with transudative effusions in the non-tuberculous group [20, 21, 24–26, 32, 33, 36, 37, 40, 42, 44, 47, 48, 50, 54, 55, 57–61]. One article described two distinct cohorts of patients, and both were considered as two separate studies for final analysis [59]. The 44 publications included in our analysis therefore provided 45 sets of paired diagnostic accuracy data for pleural fluid ADA and IFN-γ.

**Table 1:** Characteristics of studies included in data synthesis.

Primary author, publication year	Country of study	Prospective study	Case-control design	Inclusion criteria	Exclusion criteria	HIV+ patients	Transudative effusions in non-TPE group	Standard for TPE diagnosis	Definite TB (%)*
Hsu, 1989 [19]	China	Yes	No	Inpatients with effusion	NS	NS	No	MP	100
Ribera, 1990 [20]	Spain	NS	Yes	Inpatients with effusion	NS	NS	Yes	MP	100
Aoki, 1994 [21]	Japan	Yes	No	NS	NS	NS	Yes	MP	100
Jeon, 1998 [22]	Korea	Yes	Yes	NS	NS	NS	No	CMP	75.0
Kim, 1998 [23]	Korea	Yes	Yes	TPE or malignant effusion	NS	NS	No	MP	100
Zhu, 1999 [24]	China	Yes	No	NS	NS	NS	Yes	CMP	NS
Villegas, 2000 [25]	Colombia	Yes	No	Age >18 years	NS	NS	Yes	CMP	68.9
Poyraz, 2004 [26]	Turkey	Yes	Yes	Inpatients with effusion	NS	NS	Yes	P	100
El-Ansary, 2005 [27]	Egypt	Yes	No	Diagnosed pleural effusion	NS	NS	No	NS	NS
Gao, 2005 [28]	China	Yes	No	NS	NS	NS	No	CMP	NS
Okamoto, 2005 [29]	Japan	Yes	No	Lymphocytic exudative effusion	No definite diagnosis	NS	No	MP	100
Park, 2005 [30]	Korea	Yes	No	Unilateral effusion	NS	NS	No	CMP	NS
Sharma, 2005 [31]	India	Yes	No	NS	Immunosuppressive drugs, organ dysfunction, pregnancy	None	No	MP	100
Morimoto, 2006 [32]	Japan	Yes	No	NS	NS	NS	Yes	CMP	NS
Ariga, 2007 [33]	Japan	Yes	No	Definite etiology for effusion	NS	None	Yes	M	100
Daniil, 2007 [34]	Greece	Yes	No	NS	NS	NS	No	MP	100
Xue, 2007 [35]	China	Yes	Yes	Confirmed TPE or malignant effusion	NS	NS	No	MP	100
Krenke, 2008 [36]	Poland	Yes	No	Inpatients with effusion	No definite diagnosis	NS	Yes	MP	100
Titarenko, 2008 [37]	Russia	Yes	No	NS	NS	NS	NS	NS	NS
Dheda, 2009 [38]	South Africa	Yes	No	Suspected TPE	Inadequate fluid sample	26/56	No	CMP	87.3
Valdes, 2009 [39]	Spain	Yes	No	Inpatients with effusion	NS	NS	No	MP	100
Wu, 2010 [40]	China	No	No	Definitely diagnosed effusion	NS	NS	Yes	CMP	91.3
Ambade, 2011 [41]	India	Yes	No	Inpatients with effusion	Incomplete data	NS	No	MP	100
Kalantri, 2011 [42]	India	Yes	No	NS	NS	NS	Yes	CMP	32.5
Liu, 2011 [43]	Taiwan	Yes	No	Inpatients with lymphocytic exudative effusion due to TB or malignancy	NS	NS	No	CMP	NS
Wang, 2012 [44]	China	Yes	No	Inpatients with effusion	NS	NS	Yes	CMP	85.9
Keng, 2013 [45]	Taiwan	Yes	No	Lymphocytic exudative effusion	NS	NS	No	CMP	96.8
Khan, 2013 [46]	Qatar	Yes	No	Inpatients with effusion	NS	NS	No	MP	100
Lee, 2013 [47]	Korea	Yes	Yes	Inpatients with effusion	No definite diagnosis, inadequate sample	NS	Yes	MP	100
Wu, 2013 [48]	China	Yes	No	Inpatients with effusion of definite etiology	NS	None	Yes	CMP	80
Li, 2014 [49]	China	Yes	No	TPE or malignant effusion	No definite diagnosis	NS	No	MP	100
Valdes, 2014 [50]	Spain	Yes	No	Inpatients with effusion	No definite diagnosis	NS	Yes	MP	100
Yurt, 2014 [51]	Turkey	Yes	No	Inpatients with effusion	HIV seropositive, empyema, transudate, taking ATT, no definite diagnosis	None	No	MP	100
Ali, 2015 [52]	Egypt	Yes	Yes	Confirmed TB or malignancy	NS	None	No	MP	100
Dong, 2015 [53]	China	Yes	Yes	TPE or malignant	NS	NS	No	CMP	NS
Klimiuk, 2015 [54]	Poland	Yes	No	Newly diagnosed effusion	Inadequate data, no definite diagnosis	NS	Yes	MP	100
Shu, 2015 [55]	Taiwan	Yes	No	Lymphocytic exudative effusion	HIV seropositive	None	NS	MP	100
Jethani, 2016 [56]	India	Yes	Yes	Exudative effusion, age >20 years	Empyema, hemothorax, transudate, HIV seropositive	None	No	CMP	NS
Chung, 2017 [57]	Korea	Yes	No	Inpatients with effusion, age > = 18 years	Inadequate sample	NS	Yes	CMP	81.1
Santos, 2018 [58]	Brazil	Yes	No	Patients aged > = 18 years with effusion	Pregnancy	4/60	Yes	CMP	48.5
Wang, 2018 [59]	China	Yes	No	Inpatients with effusion of known etiology	NS	NS	NS	MP	100
Faria, 2019 [60]	Brazil	Yes	No	NS	NS	NS	Yes	MP	100
Li, 2019 [61]	China	Yes	Yes	Patients with effusion	NS	None	Yes	CMP	NS
Zhang, 2020 [62]	China	Yes	Yes	Patients aged > = 18 years with confirmed TPE or malignant effusion	Transudative effusions, heart/renal/liver failure, nephrotic syndrome, cirrhosis	NS	No	MP	100

ATT Anti-tubercular treatment, HIV Human immunodeficiency virus, NS Not specified, TB Tuberculosis, TPE Tuberculous pleural effusion

Standard for diagnosis: C Clinical, M Microbiologic, P Pathologic

* Definite TB implies microbiologic and/or histopathologic confirmation of diagnosis in patients with tuberculous pleural effusion

Overall, 2036 patients of TPE and 2937 patients with other pleural effusions were evaluated with ADA, and 2036 patients of TPE and 2938 patients with other pleural effusions were evaluated with IFN-γ, in the 45 datasets included for analysis. Most studies (29, 64.4%) used the Guisti method for the ADA assay, while 13 (28.9%) studies used other procedures (S2 Table) [21, 28, 29, 32, 35, 40, 49, 50, 57, 59, 61, 62]. Three (6.7%) studies did not report the ADA assay technique [30, 33, 54]. The IFN-γ assay was performed using enzyme-linked immunosorbent assay (ELISA) in most (40, 88.9%) studies. Four (8.9%) studies used another method, and one (2.2%) study did not specify the assay technique (S3 Table) [20, 34, 43, 60, 62]. Three studies reported data on two or more diagnostic thresholds for the assays (S2 Table) [23, 32, 45]. Diagnostic thresholds for ADA varied widely between 15.5–70 IU/L. The variability was even greater for IFN-γ thresholds, which were reported as either weight or activity per unit volume (S2 Table). Two (4.5%) studies did not specify the IFN-γ threshold used for diagnosis [34, 42]. Only one of two cohorts from one study showed overall good quality, with no risk of bias across all the QUADAS-2 domains [59]. High risk of bias was observed in all other studies (S1 Fig), which was primarily related to lack of blinding and/or use of pre-specified diagnostic thresholds. Eleven (24.4%) studies also showed applicability concerns in the patient selection domain. There was no publication bias (S2 Fig).

### Individual test diagnostic accuracy

S3 Table provides the diagnostic accuracy estimates calculated from individual studies. There was substantial heterogeneity between the included studies (*I*^2^ 94.8% for ADA and 80.6% for IFN-γ respectively). The sensitivity of ADA for diagnosis of TPE varied from 0.40 to 1.00, and specificity from 0.68 to 1.00 (S3 Fig). The summary sensitivity and specificity were 0.88 (95% CI 0.85–0.91) and 0.91 (95% CI 0.89–0.92) respectively. The sensitivity of IFN-γ for diagnosis of TPE varied from 0.61 to 1.00, and specificity from 0.68 to 1.00. The summary sensitivity and specificity were both superior to values for ADA at 0.91 (95% CI 0.89–0.94) and 0.96 (95% CI 0.94–0.97) respectively. The summary positive and negative likelihood ratios were 9.47 (95% CI 7.79–11.51) and 0.13 (95% CI 0.10–0.16) for ADA, and 21.13 (95% CI 14.75–30.29) and 0.09 (95% CI 0.07–0.12) for IFN-γ, respectively. A higher summary positive likelihood ratio (above 10) and a lower summary negative likelihood ratio (below 0.1) indicated that IFN-γ was better than ADA at both confirming as well as excluding a diagnosis of TPE (S4 Fig). Subgroup analysis did not indicate any noticeable improvement in diagnostic accuracy for any category of the prespecified covariates (S4 Table).

### Comparative test diagnostic accuracy

**Fig 2:**
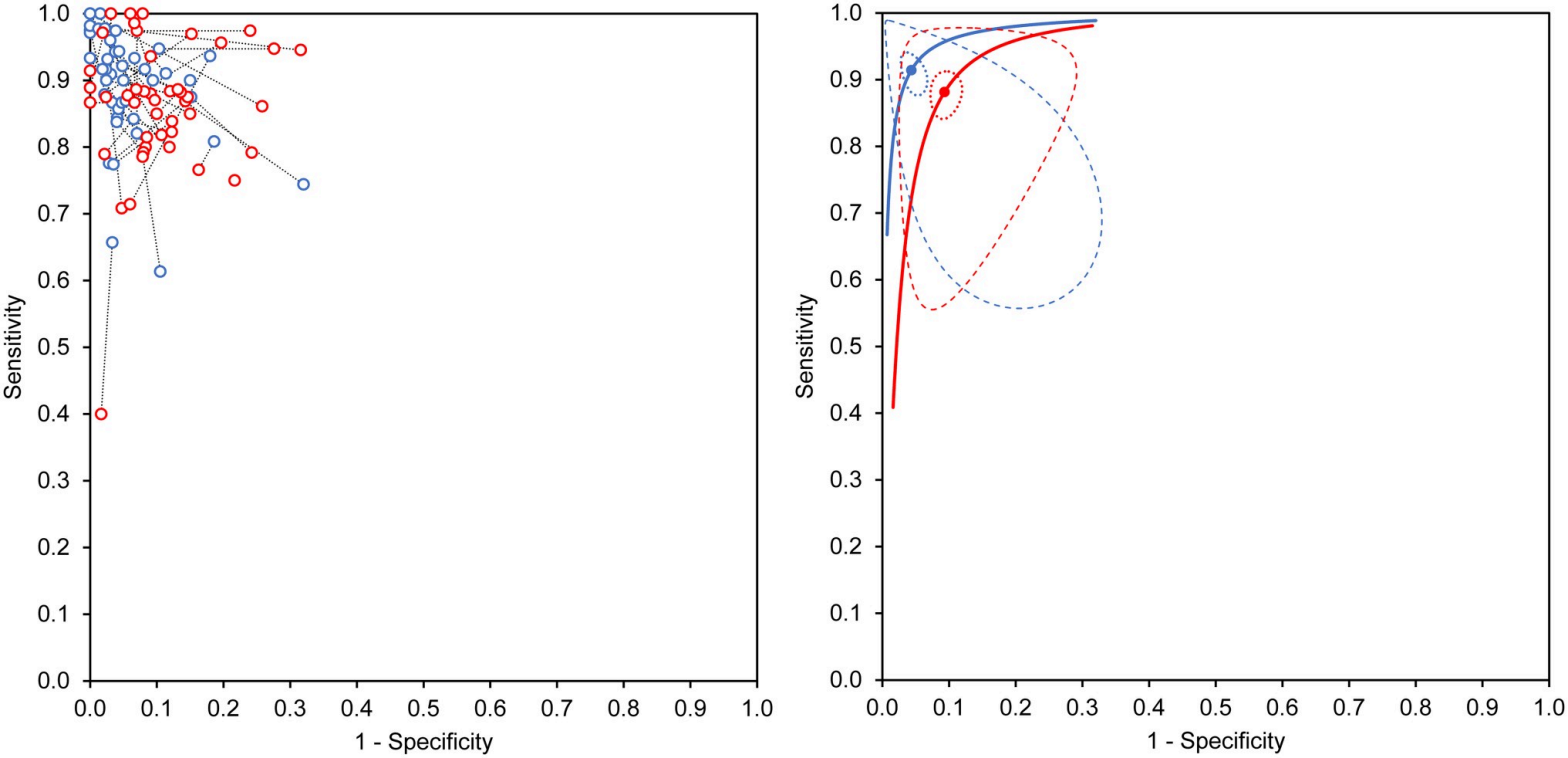
Comparison of summary points and summary curves, for studies evaluating both pleural fluid adenosine deaminase (red) and interferon-gamma (blue), in receiver operating characteristic (ROC) space. The left panel shows pairs of accuracy estimates from each included study, joined together by dotted lines. The right panel shows individual hierarchical summary ROC (HSROC) plots for the two tests, with the solid circles indicating the summary diagnostic accuracy points. The dotted ellipses represent 95% confidence regions around these summary estimates. The dashed lines represent the 95% prediction region (area within which one is 95% certain the results of a new study will lie).

The individual HSROC plots for ADA and IFN-γ assays (Fig 2) appeared symmetrical (shape parameter beta for plots for ADA and IFN-γ were -0.25, p = 0.35, and 0.11, p = 0.61 respectively) implying that test accuracy for either assay was not dependent on test threshold. The two plots did not cross each other, and the plot for IFN-γ was positioned more towards the desired upper left corner of the graph, suggesting that pleural fluid IFN-γ was consistently more accurate than ADA for diagnosis of TPE across the entire range of observations from the included studies. The 95% confidence ellipses around the summary diagnostic accuracy estimates for the two tests were narrow did not overlap, implying that on direct comparison, IFN-γ had a significantly better diagnostic accuracy than ADA (Fig 2). The 95% prediction regions for both tests were much wider and overlapping, again indicative of substantial heterogeneity.

HSROC meta-regression, allowing the test type covariate (ADA or IFN-γ) to influence accuracy, shape, and threshold, showed improvement in model fit as compared to a model without adjustment for covariate (reduction in -2 log likelihood ratio from 944.8 to 909.0, p <0.001 at three degrees of freedom). We then fitted a less complex model based on common HSROC plot shape, by allowing the test type to influence only accuracy and threshold. This did not significantly improve model fit further (change in -2 log likelihood ratio from 909.0 to 911.3, p = 0.13 at one degree of freedom), indicating lack of statistical evidence of a difference in shape of plots for the two tests (S5 Table and S4 Fig). Since the individual plots were symmetrical and showed similar underlying shape, RDOR obtained from this model was considered the summary measure of relative accuracy. Based on this model, the diagnosis of TPE using IFN-γ assay showed higher accuracy (p <0.001) than diagnosis using ADA assay (RDOR 2.22, 95% CI 1.68–2.94). Both relative sensitivity (1.03, 95% CI 1.01–1.05) and relative specificity (1.04, 95% CI 1.02–1.05) values suggested better diagnostic performance for IFN-γ as compared to ADA. In absolute terms, both summary sensitivity and summary specificity were higher by 0.03 for IFN-γ as compared to ADA (S5 Table). This means that, as compared to ADA, IFN-γ will detect an additional 3% patients with TPE and will exclude this diagnosis among 3% additional patients without TPE. Further attempts at simplification of the model by allowing test type to influence only accuracy did not significantly alter model fit, implying that the meta-regression results were not significantly influenced by test threshold (S5 Table and S5 Fig).

### Grading of evidence

Overall, we found moderate grade evidence regarding diagnostic accuracy of pleural fluid ADA and TNF for TPE diagnosis (Table 2). On evaluating the clinical implications of applying either index test in a hypothetical cohort of 1000 patients having various levels of TPE prevalence, IFN-γ performed better than ADA. Based on our summary diagnostic accuracy estimates, IFN-γ is likely to miss about 30% less TPE patients as compared to ADA in relative terms across a range of pre-test probabilities. Similarly, IFN-γ testing is likely reduce false positive results by more than half across a range of pre-test probabilities (Table 1).

**Table 2:** Summary of findings from studies evaluating pleural fluid adenosine deaminase (ADA) and unstimulated pleural fluid interferon-gamma (IFN-γ) for diagnosing pleural tuberculosis.

Outcome	Number of studies and number of patients	Study design	Factors that may decrease certainty of evidence			Effect per 1000 patients tested			Certainty of evidence for test accuracy
			Risk of bias	Indirectness Inconsistency Imprecision	Publication bias	5% pre-test probability	25% pre-test probability	50% pre-test probability	
ADA									
True positives	45 studies 2036 patients	Cohort and case-control studies	Serious*	Not serious	None	44 (43 to 45)	220 (213 to 227)	441 (426 to 453)	⨁⨁⨁**◯** MODERATE
False negatives						6 (5 to 7)	30 (23 to 37)	59 (47 to 74)	
True negatives	45 studies 2937 patients	Cohort and case-control studies	Serious*	Not serious	None	862 (843 to 878)	680 (665 to 693)	453 (444 to 462)	⨁⨁⨁**◯** MODERATE
False positives						88 (72 to 107)	70 (57 to 85)	47 (38 to 56)	
IFN-γ									
True positives	45 studies 2036 patients	Cohort and case-control studies	Serious*	Not serious	None	46 (44 to 47)	229 (221 to 234)	457 (443 to 468)	⨁⨁⨁**◯** MODERATE
False negatives						4 (3 to 6)	21 (16 to 29)	43 (32 to 57)	
True negatives	45 studies 2938 patients	Cohort and case-control studies	Serious*	Not serious	None	909 (892 to 921)	718 (704 to 727)	478 (470 to 485)	⨁⨁⨁**◯** MODERATE
False positives						41 (29 to 58)	32 (23 to 46)	22 (15 to 30)	

* Most studies had no blinding, and/or did not use pre-specified diagnostic thresholds

## Discussion

We analyzed 45 paired datasets (4974 patients) evaluating the performance of pleural fluid ADA and IFN-γ from 44 publications. These studies used different diagnostic thresholds and reference standards for diagnosing TPE. Our findings suggest that both ADA and IFN-γ show good summary diagnostic accuracy as individual tests. On direct comparison using HSROC meta-regression, IFN-γ showed better summary accuracy for TPE diagnosis as compared to ADA (RDOR 2.22, 95% CI 1.68–2.94) without any threshold effect.

The summary diagnostic accuracy estimates derived from our study, for both pleural fluid ADA and IFN-γ, are largely similar to observations from our recent larger meta-analyses evaluating these assays individually [2, 3]. The main strength of our analysis is the large sample size of paired data on the two index tests that was available for direct comparison, allowing us to provide robust comparative diagnostic accuracy estimates. We preferred the Rutter and Gatsonis HSROC model over a bivariate model to accommodate the substantial variability in diagnostic thresholds for both ADA and IFN-γ between the included studies. The positioning of HSROC plots, as well as the numerical data for summary estimates, suggests pleural fluid IFN-γ to be a better diagnostic marker for TPE than pleural fluid ADA. At present, pleural fluid ADA is favoured as the investigation of choice while evaluating patients suspected to have TPE, especially when a definite microbiological diagnosis is not forthcoming. Our results suggest pleural fluid IFN-γ to be a better discriminator, both for confirmation and exclusion of TPE, in everyday clinical practice (Table 2). This information is likely to influence current algorithms for evaluating patients with pleural effusion, especially with the development of a low-cost ultrasensitive rapid immuno-suspension test that could lead to wider deployment of pleural fluid IFN-γ assays [63]. A formal cost-effectiveness analysis is, however, beyond the scope of our review. Some consensus is still needed to define a threshold value clinically useful in diverse settings [3].

Our analysis has few limitations. The studies included in this review showed substantial heterogeneity. Almost all studies showed high risk of bias, and several had high concern regarding applicability. This reduces the strength of validity and applicability of our observations. Another major limitation was the diversity of assay techniques and diagnostic thresholds used in the studies, which we could not incorporate within our analysis. Several investigators ‘adapted’ the Guisti method of ADA estimation in their laboratories, and this may have influenced the variability in ADA thresholds and results to some extent. Although our HSROC models did not suggest any threshold effect, we are unable to comment on absolute or relative test accuracy at any specific pair of thresholds that could be clinically recommended. In addition, many individual studies had several limitations. Half of the publications had enrolled patients with transudative pleural effusion, which could have artificially increased specificity estimates since TPE is a diagnostic consideration in exudative effusions only. Most studies did not pre-specify a diagnostic threshold aimed at either confirming or excluding TPE, but rather estimated a reasonable trade-off from post-hoc ROC analysis. We have summarized the diagnostic performance of ADA and IFN-γ as isolated investigations but cannot comment if their combination, or concurrent use with results of other investigations, can further improve their role in routine clinical decision-making. There is limited data to suggest that combining pleural fluid ADA and IFN-γ for diagnosis of TPE can improve specificity to up to 100% at the expense of some reduction in sensitivity [25, 45, 58].

## Conclusion

In conclusion, the findings from our meta-analysis indicate that pleural fluid IFN-γ estimation has better diagnostic accuracy than pleural fluid ADA estimation for the diagnosis of TPE. We believe that pleural fluid IFN-γ is likely be used as a primary diagnostic biomarker while evaluating patients with suspected TPE.

## Supporting information

S1 Table Reasons for excluding studies on full-text review. (PDF)

S2 Table Pleural fluid assays for the index tests, and their results, from the studies included in data synthesis. (PDF)

S3 Table Diagnostic accuracy estimates for pleural fluid unstimulated interferon-gamma and adenosine deaminase assays. (PDF)

S4 Table Evaluation of factors affecting individual summary diagnostic accuracy estimates of pleural fluid assays. (PDF)

S5 Table Parameters and summary estimates from hierarchical summary receiver operating characteristic models. (PDF)

S1 Fig Risk of bias and applicability concerns summary. (PDF)

S2 Fig Publication bias. (PDF)

S3 Fig Coupled forest plot from studies on diagnostic accuracy of pleural fluid adenosine deaminase and interferon-gamma in the same patient population. (PDF)

S4 Fig Likelihood ratio matrix. (PDF)

S5 Fig Summary plots from hierarchical summary receiver operating characteristic modeling. (PDF)

## References

[1] Shaw JA, Ahmed L, Koegelenberg CFN. Effusions related to TB. ERS Monograph. 2020;2020(9781849841160):172–92.

[2] Aggarwal AN, Agarwal R, Sehgal IS, Dhooria S. Adenosine deaminase for diagnosis of tuberculous pleural effusion: A systematic review and meta-analysis. PLoS One. 2019;14(3):e0213728. Epub 2019/03/27. doi: 10.1371/journal.pone.0213728 . 30913213 PMC6435228

[3] Aggarwal AN, Agarwal R, Dhooria S, Prasad KT, Sehgal IS, Muthu V. Unstimulated pleural fluid interferon-gamma for diagnosis of tuberculous pleural effusion: a systematic review and meta-analysis. J Clin Microbiol. 2020. Epub 2020/11/20. doi: 10.1128/JCM.02112-20 . 33208475 PMC8091832

[4] Greco S, Girardi E, Masciangelo R, Capoccetta GB, Saltini C. Adenosine deaminase and interferon gamma measurements for the diagnosis of tuberculous pleurisy: a meta-analysis. Int J Tuberc Lung Dis. 2003;7(8):777–86. Epub 2003/08/19. . 12921155

[5] Takwoingi Y, Leeflang MM, Deeks JJ. Empirical evidence of the importance of comparative studies of diagnostic test accuracy. Ann Intern Med. 2013;158(7):544–54. Epub 2013/04/03. doi: 10.7326/0003-4819-158-7-201304020-00006 . 23546566

[6] Macaskill P, Gatsonis C, Deeks JJ, Harbord RM, Takwoingi Y. Chapter 10: Analysing and presenting results. In: Deeks JJ, Bossuyt PM, Gatsonis C, editors. Handbook for Systematic Reviews of Diagnostic Test Accuracy, version 10: The Cochrane Collaboration; 2010.

[7] Skouras VS, Kalomenidis I. Pleural fluid tests to diagnose tuberculous pleuritis. Curr Opin Pulm Med. 2016;22(4):367–77. Epub 2016/04/12. doi: 10.1097/MCP.0000000000000277 . 27064428

[8] Liberati A, Altman DG, Tetzlaff J, Mulrow C, Gotzsche PC, Ioannidis JP, et al. The PRISMA statement for reporting systematic reviews and meta-analyses of studies that evaluate health care interventions: explanation and elaboration. Ann Intern Med. 2009;151(4):W65–94. doi: 10.7326/0003-4819-151-4-200908180-00136 . 19622512

[9] Salameh JP, Bossuyt PM, McGrath TA, Thombs BD, Hyde CJ, Macaskill P, et al. Preferred reporting items for systematic review and meta-analysis of diagnostic test accuracy studies (PRISMA-DTA): explanation, elaboration, and checklist. BMJ. 2020;370:m2632. Epub 2020/08/21. doi: 10.1136/bmj.m2632 . 32816740

[10] Clopper CJ, Pearson ES. The use of confidence or fiducial limits illustrated in the case of the binomial. Biometrika. 1934;26(4):404–13.

[11] Rutter CM, Gatsonis CA. A hierarchical regression approach to meta-analysis of diagnostic test accuracy evaluations. Stat Med. 2001;20(19):2865–84. doi: 10.1002/sim.942 . 11568945

[12] Takwoingi Y, Partlett C, Riley RD, Hyde C, Deeks JJ. Methods and reporting of systematic reviews of comparative accuracy were deficient: a methodological survey and proposed guidance. J Clin Epidemiol. 2020;121:1–14. Epub 2019/12/18. doi: 10.1016/j.jclinepi.2019.12.007 . 31843693 PMC7203546

[13] Bossuyt P, Davenport C, Deeks J, Hyde C, Leeflang M, Scholten R. Chapter 11: Interpreting results and drawing conclusions. In: Deeks JJ, Bossuyt PM, Gatsonis C, editors. Handbook for Systematic Reviews of Diagnostic Test Accuracy, version 09: The Cochrane Collaboration; 2013.

[14] Whiting PF, Rutjes AW, Westwood ME, Mallett S, Deeks JJ, Reitsma JB, et al. QUADAS-2: a revised tool for the quality assessment of diagnostic accuracy studies. Ann Intern Med. 2011;155(8):529–36. doi: 10.7326/0003-4819-155-8-201110180-00009 . 22007046

[15] Higgins JP, Thompson SG, Deeks JJ, Altman DG. Measuring inconsistency in meta-analyses. BMJ. 2003;327(7414):557–60. doi: 10.1136/bmj.327.7414.557 . 12958120 PMC192859

[16] World Health Organization. Global Tuberculosis Report 2019. Geneva: WHO Press; 2019.

[17] Schünemann H, Brożek J, Guyatt G, Oxman A, eds. Handbook for grading the quality of evidence and the strength of recommendations using the GRADE approach (updated October 2013). GRADE Working Group, 2013. https://gdt.guidelinedevelopment.org/app/handbook/handbook.html.

[18] Takwoingi Y, Deeks JJ. MetaDAS: A SAS macro for meta-analysis of diagnostic accuracy studies (version 1.3, July 2010). https://methods.cochrane.org/sdt/software-meta-analysis-dta-studies.

[19] Hsu WH, Chiang CD, Chen WT, Chen CF. Diagnostic value of adenosine deaminase and gamma-interferon in tuberculous and malignant pleural effusions. Taiwan Yi Xue Hui Za Zhi. 1989;88(9):879–82. Epub 1989/09/01. . 2516112

[20] Ribera E, Martínez-Vázquez JM, Ocaña I, Ruiz I, Segura RM, Encabo G, et al. Gamma interferon and adenosine deaminase in pleuritis. Med Clin (Barc). 1990;94(10):364–7. Epub 1990/03/17. . 2110605

[21] Aoki Y, Katoh O, Nakanishi Y, Kuroki S, Yamada H. A comparison study of IFN-gamma, ADA, and CA125 as the diagnostic parameters in tuberculous pleuritis. Respir Med. 1994;88(2):139–43. Epub 1994/02/01. doi: 10.1016/0954-6111(94)90027-2 . 8146413

[22] Jeon DS, Yun SM, Park SS, Lee HJ, Kim YS, Lee MK, et al. The significance of IL-10, IL-12, IFN-γ and ADA in tuberculous pleural fluid. Tuber Respir Dis. 1998;45(2):301–10.

[23] Kim MS, Yang SE, Chi HS, Kim WS, Kim WD. The usefulness of pleural IFN-γ level in differential diagnosis of tuberculous pleural effusion and malignant pleural effusion. Tuber Respir Dis. 1998;45(2):280–9.

[24] Zhu X, Zhang J, Zheng X, Wu Z, Pan Y. Investigation on differential diagnostic value of multi-index detection of pleural effusion. Zhongguo Fei Ai Za Zhi. 1999;2(2):77–9. Epub 1999/12/25. doi: 10.3779/j.issn.1009-3419.1999.02.05 . 20929634

[25] Villegas MV, Labrada LA, Saravia NG. Evaluation of polymerase chain reaction, adenosine deaminase, and interferon-gamma in pleural fluid for the differential diagnosis of pleural tuberculosis. Chest. 2000;118(5):1355–64. Epub 2000/11/18. doi: 10.1378/chest.118.5.1355 . 11083686

[26] Poyraz B, Kaya A, Ciledağ A, Oktem A, Gönüllü U. Diagnostic significance of gamma-interferon in tuberculous pleurisy. Tuberk Toraks. 2004;52(3):211–7. Epub 2004/09/08. . 15351932

[27] El-Ansary AK, Radwan MA. Evaluation of cytokines in pleural fluid for the differential diagnosis of tuberculous pleurisy. Biochim Clin. 2005;29(1):13–20.

[28] Gao ZC, Tian RX. Clinical investigation on diagnostic value of interferon-gamma, interleukin-12 and adenosine deaminase isoenzyme for tuberculous pleurisy. Chin Med J (Engl). 2005;118(3):234–7. Epub 2005/03/03. . 15740654

[29] Okamoto M, Kawabe T, Iwasaki Y, Hara T, Hashimoto N, Imaizumi K, et al. Evaluation of interferon-gamma, interferon-gamma-inducing cytokines, and interferon-gamma-inducible chemokines in tuberculous pleural effusions. J Lab Clin Med. 2005;145(2):88–93. Epub 2005/03/05. doi: 10.1016/j.lab.2004.11.013 . 15746651

[30] Park JS, Kim YS, Jee YK, Lee KY, Choi J, Cho S, et al. The utility of pleural fluid cell IFN-γ production assay in the diagnosis of tuberculous pleurisy. Tuber Respir Dis. 2005;59(2):186–92. doi: 10.4046/trd.2005.59.2.186

[31] Sharma SK, Banga A. Pleural fluid interferon-gamma and adenosine deaminase levels in tuberculosis pleural effusion: a cost-effectiveness analysis. J Clin Lab Anal. 2005;19(2):40–6. Epub 2005/03/10. doi: 10.1002/jcla.20054 . 15756707 PMC6808038

[32] Morimoto T, Takanashi S, Hasegawa Y, Fujimoto K, Okudera K, Hayashi A, et al. Level of antibodies against mycobacterial glycolipid in the effusion for diagnosis of tuberculous pleural effusion. Respir Med. 2006;100(10):1775–80. Epub 2006/03/17. doi: 10.1016/j.rmed.2006.01.023 . 16540297

[33] Ariga H, Kawabe Y, Nagai H, Kurashima A, Masuda K, Matsui H, et al. Diagnosis of active tuberculous serositis by antigen-specific interferon-gamma response of cavity fluid cells. Clin Infect Dis. 2007;45(12):1559–67. Epub 2008/01/15. doi: 10.1086/523591 . 18190316

[34] Daniil ZD, Zintzaras E, Kiropoulos T, Papaioannou AI, Koutsokera A, Kastanis A, et al. Discrimination of exudative pleural effusions based on multiple biological parameters. Eur Respir J. 2007;30(5):957–64. Epub 2007/08/11. doi: 10.1183/09031936.00126306 . 17690119

[35] Xue K, Xiong S, Xiong W. Clinical value of vascular endothelial growth factor combined with interferon-gamma in diagnosing malignant pleural effusion and tuberculous pleural effusion. J Huazhong Univ Sci Technolog Med Sci. 2007;27(5):495–7. Epub 2007/12/07. doi: 10.1007/s11596-007-0504-4 . 18060618

[36] Krenke R, Safianowska A, Paplinska M, Nasilowski J, Dmowska-Sobstyl B, Bogacka-Zatorska E, et al. Pleural fluid adenosine deaminase and interferon gamma as diagnostic tools in tuberculosis pleurisy. J Physiol Pharmacol. 2008;59 Suppl 6:349–60. Epub 2009/02/28. . 19218659

[37] Titarenko OT, Esmedliaeva DS, D’Iakova M E, Perova TL, Popov M. Gamma-interferon and adenosine deaminase in the diagnosis of tuberculous pleurisy. Probl Tuberk Bolezn Legk. 2008;(12):27–30. Epub 2009/02/21. . 19227321

[38] Dheda K, van Zyl-Smit RN, Sechi LA, Badri M, Meldau R, Meldau S, et al. Utility of quantitative T-cell responses versus unstimulated interferon-{gamma} for the diagnosis of pleural tuberculosis. Eur Respir J. 2009;34(5):1118–26. Epub 2009/04/24. doi: 10.1183/09031936.00005309 . 19386693

[39] Valdés L, San José E, Alvarez Dobaño JM, Golpe A, Valle JM, Penela P, et al. Diagnostic value of interleukin-12 p40 in tuberculous pleural effusions. Eur Respir J. 2009;33(4):816–20. Epub 2008/12/03. doi: 10.1183/09031936.00085008 . 19047317

[40] Wu SH, Li CT, Lin CH, Chu JJ, Cheng ML, Lin KH. Soluble Fas ligand is another good diagnostic marker for tuberculous pleurisy. Diagn Microbiol Infect Dis. 2010;68(4):395–400. Epub 2010/10/12. doi: 10.1016/j.diagmicrobio.2010.08.008 . 20926222

[41] Ambade V, Arora MM, Rai SP, Nikumb SK, Basannar DR. Markers for differentiation of tubercular pleural effusion from non-tubercular effusion. Med J Armed Forces India. 2011;67(4):338–42. doi: 10.1016/S0377-1237(11)60080-4 27365843 PMC4920659

[42] Kalantri Y, Hemvani N, Chitnis DS. Evaluation of real-time polymerase chain reaction, interferon-gamma, adenosine deaminase, and immunoglobulin A for the efficient diagnosis of pleural tuberculosis. Int J Infect Dis. 2011;15(4):e226–31. Epub 2011/01/14. doi: 10.1016/j.ijid.2010.11.011 . 21227729

[43] Liu YC, Shin-Jung Lee S, Chen YS, Tu HZ, Chen BC, Huang TS. Differential diagnosis of tuberculous and malignant pleurisy using pleural fluid adenosine deaminase and interferon gamma in Taiwan. J Microbiol Immunol Infect. 2011;44(2):88–94. Epub 2011/03/29. doi: 10.1016/j.jmii.2010.04.001 . 21439509

[44] Wang H, Yue J, Yang J, Gao R, Liu J. Clinical diagnostic utility of adenosine deaminase, interferon-γ, interferon-γ-induced protein of 10 kDa, and dipeptidyl peptidase 4 levels in tuberculous pleural effusions. Heart Lung. 2012;41(1):70–5. Epub 2011/09/16. doi: 10.1016/j.hrtlng.2011.04.049 . 21917315

[45] Keng LT, Shu CC, Chen JY, Liang SK, Lin CK, Chang LY, et al. Evaluating pleural ADA, ADA2, IFN-γ and IGRA for diagnosing tuberculous pleurisy. J Infect. 2013;67(4):294–302. Epub 2013/06/26. doi: 10.1016/j.jinf.2013.05.009 . 23796864

[46] Khan FY, Hamza M, Omran AH, Saleh M, Lingawi M, Alnaqdy A, et al. Diagnostic value of pleural fluid interferon-gamma and adenosine deaminase in patients with pleural tuberculosis in Qatar. Int J Gen Med. 2013;6:13–8. Epub 2013/02/05. doi: 10.2147/IJGM.S39345 . 23378780 PMC3553648

[47] Lee KS, Kim HR, Kwak S, Choi KH, Cho JH, Lee YJ, et al. Association between elevated pleural interleukin-33 levels and tuberculous pleurisy. Ann Lab Med. 2013;33(1):45–51. Epub 2013/01/10. doi: 10.3343/alm.2013.33.1.45 . 23301222 PMC3535196

[48] Wu YB, Ye ZJ, Qin SM, Wu C, Chen YQ, Shi HZ. Combined detections of interleukin 27, interferon-γ, and adenosine deaminase in pleural effusion for diagnosis of tuberculous pleurisy. Chin Med J (Engl). 2013;126(17):3215–21. Epub 2013/09/17. . 24033939

[49] Li M, Wang H, Wang X, Huang J, Wang J, Xi X. Diagnostic accuracy of tumor necrosis factor-alpha, interferon-gamma, interleukin-10 and adenosine deaminase 2 in differential diagnosis between tuberculous pleural effusion and malignant pleural effusion. J Cardiothorac Surg. 2014;9:118. Epub 2014/07/06. doi: 10.1186/1749-8090-9-118 . 24984978 PMC4227019

[50] Valdés L, San José E, Ferreiro L, Golpe A, Gude F, Álvarez-Dobaño JM, et al. Interleukin 27 could be useful in the diagnosis of tuberculous pleural effusions. Respir Care. 2014;59(3):399–405. Epub 2013/08/22. doi: 10.4187/respcare.02749 . 23962500

[51] Yurt S, Küçükergin C, Yigitbas BA, Seçkin S, Tigin HC, Koşar AF. Diagnostic utility of serum and pleural levels of adenosine deaminase 1–2, and interferon-γ in the diagnosis of pleural tuberculosis. Multidiscip Respir Med. 2014;9(1):12. Epub 2014/03/08. doi: 10.1186/2049-6958-9-12 . 24602306 PMC3978112

[52] Ali AHK, Mahmoud TM, Ahmed H. Differential diagnostic efficiency of T cells subsets versus interferon-gamma, tumor necrosis factor-alpha and adenosine deaminase in distinguishing tuberculous from malignant pleural effusions. Egyptian J Chest Dis Tuber. 2015;64(3):645–51. doi: 10.1016/j.ejcdt.2015.03.005

[53] Dong YK, Li AZ, Zheng LH, Chi YP, Wang YH, Wang QM, et al. The value of adenosine deaminase, interferon-gamma, and interferon-gamma induced protein of 10KD in the diagnosis of tuberculous pleuritis. Med J Chin People’s Liberation Army. 2015;40(6):458–62.

[54] Klimiuk J, Krenke R, Safianowska A, Korczynski P, Chazan R. Diagnostic performance of different pleural fluid biomarkers in tuberculous pleurisy. Adv Exp Med Biol. 2015;852:21–30. Epub 2014/12/20. doi: 10.1007/5584_2014_105 . 25523627

[55] Shu CC, Wang JY, Hsu CL, Keng LT, Tsui K, Lin JF, et al. Diagnostic role of inflammatory and anti-inflammatory cytokines and effector molecules of cytotoxic T lymphocytes in tuberculous pleural effusion. Respirology. 2015;20(1):147–54. Epub 2014/10/31. doi: 10.1111/resp.12414 . 25355638

[56] Jethani V, Sindhwani G, Mehrotra V, Kotwal A, Khanduri R. Adenosine deaminase and interferon-gamma in diagnosis of tubercular pleural effusion. Int J Res Med Sci. 2016;4(9):3951–5.

[57] Chung W, Jung Y, Lee K, Park J, Sheen S, Park K. CXCR3 ligands in pleural fluid as markers for the diagnosis of tuberculous pleural effusion. Int J Tuberc Lung Dis. 2017;21(12):1300–6. Epub 2018/01/04. doi: 10.5588/ijtld.17.0232 . 29297451

[58] Santos AP, Corrêa RDS, Ribeiro-Alves M, Soares da Silva ACO, Mafort TT, Leung J, et al. Application of Venn’s diagram in the diagnosis of pleural tuberculosis using IFN-γ, IP-10 and adenosine deaminase. PLoS One. 2018;13(8):e0202481. Epub 2018/08/28. doi: 10.1371/journal.pone.0202481 . 30148839 PMC6110466

[59] Wang W, Zhou Q, Zhai K, Wang Y, Liu JY, Wang XJ, et al. Diagnostic accuracy of interleukin 27 for tuberculous pleural effusion: two prospective studies and one meta-analysis. Thorax. 2018;73(3):240–7. Epub 2017/08/28. doi: 10.1136/thoraxjnl-2016-209718 . 28844060

[60] Faria DK, Faria CS, Doi D, Acencio MMP, Antonangelo L. Hybrid panel of biomarkers can be useful in the diagnosis of pleural and peritoneal effusions. Clin Chim Acta. 2019;497:48–53. Epub 2019/07/17. doi: 10.1016/j.cca.2019.07.015 . 31310745

[61] Li F, Zhang J, Hao X, Shen S, Ji K, Sun Y. Values of IL-27 and IFN-γ in pleural effusion in diagnosis of tuberculous pleuritis. J Jilin Univ Medicine Edition. 2019;45(2):353–8.

[62] Zhang J, Chen Y, He G, Jiang X, Chen P, Ouyang J. Differential diagnosis of tuberculous and malignant pleural effusions: comparison of the Th1/Th2 cytokine panel, tumor marker panel and chemistry panel. Scand J Clin Lab Invest. 2020;80(4):265–70. Epub 2020/02/29. doi: 10.1080/00365513.2020.1728784 . 32108543

[63] Meldau R, Randall P, Pooran A, Limberis J, Makambwa E, Dhansay M, et al. Same-day tools, including Xpert Ultra and IRISA-TB, for rapid diagnosis of pleural tuberculosis: a prospective observational study. J Clin Microbiol. 2019;57(9):e00614–19. Epub 2019/07/05. doi: 10.1128/JCM.00614-19 . 31270183 PMC6711909

