# Supplementary material for: Comparative accuracy of pleural fluid unstimulated interferon-gamma and adenosine deaminase for diagnosing pleural tuberculosis: A systematic review and meta-analysis
Source: PLoS One. 2021 Jun 24;16(6):e0253525. doi: 10.1371/journal.pone.0253525 (PMC8224977; doi:10.1371/journal.pone.0253525)
Supplement: S1 Table — (PDF) [file pone.0253525.s001.pdf]

**S1 Table.** Reasons for excluding studies on full-text review.

| | |
|---|---|
| 1. Shimokata K, Saka H, Murate T, Hasegawa Y, Hasegawa T. Cytokine content in pleural effusion. Comparison between tuberculous and carcinomatous pleurisy. Chest 1991;99:1103-7. | Only descriptive analysis |
| 2. Yokoyama A, Maruyama M, Ito M, Kohno N, Hiwada K, Yano S. Interleukin 6 activity in pleural effusion; Its diagnostic value and thrombopoietic activity. Chest 1992;102:1055-9. | Not primarily focused on either index test |
| 3. Valdés L, San José E, Alvarez D, Sarandeses A, Pose A, Chomón B, et al. Diagnosis of tuberculous pleurisy using the biologic parameters adenosine deaminase, lysozyme, and interferon gamma. Chest 1993;103:458-65. | Both index tests performed on <95% patients |
| 4. Ogawa K, Koga H, Yang B, Fukuda M, Ohno H, Yamamoto Y, et al. Differential diagnosis of tuberculous pleurisy by the measurement of cytokine concentration in pleural effusion. Kekkaku 1996;71:663-9. | Only descriptive analysis |
| 5. Hyun Joo N, Seog Chea P, Kwang Won K, Hyeong Kwan P, Young Chul K, In Seon C, et al. Diagnostic significance of TNF-alpha in tuberculous and non-tuberculous pleural effusion. Tuber Respir Dis 1997;44:611-20. | Evaluating only one of the two index tests |
| 6. Kim YC, Park KO, Bom HS, Lim SC, Park HK, Na HJ, et al. Combining ADA, protein and IFN-gamma best allows discrimination between tuberculous and malignant pleural effusion. Korean J Intern Med 1997;12:225-31. | Both index tests performed on <95% patients |
| 7. Naito T, Ohtsuka M, Ishikawa H, Satoh H, Hasegawa S. Clinical significance of cytokine measurement in pleural effusion. Kekkaku 1997;72:565-72. | Both index tests performed on <95% patients |
| 8. Ogawa K, Koga H, Hirakata Y, Tomono K, Tashiro T, Kohno S. Differential diagnosis of tuberculous pleurisy by measurement of cytokine concentrations in pleural effusion. Tuber Lung Dis 1997;78:29-34. | Both index tests performed on <95% patients |
| 9. Montes Santiago J, Gambón Deza F, Pacheco Carracedo M, Cerdá Mota T. Lymphocyte activation in tuberculous pleuritis. Correlation with adenosine deaminase (ADA), peripheral blood lymphocytes, T cell receptor subfamilies, radiographic extension and levels of Il-6 and soluble Il-2 receptor. Anales de Medicina Interna 1998;15:70-4. | No data for non-tuberculous effusions |
| 10. Valdés L, Alvarez D, San José E, Penela P, Valle JM, García-Pazos JM, et al. Tuberculous pleurisy: a study of 254 patients. Arch Intern Med 1998;158:2017-21. | No data for non-tuberculous effusions |
| 11. Oshikawa K, Sugiyama Y. Elevated soluble CD26 levels in patients with tuberculous pleurisy. Int J Tuberc Lung Dis 2001;5:868-72. | Only descriptive analysis |
| 12. Yamada Y, Nakamura A, Hosoda M, Kato T, Asano T, Tonegawa K, et al. Cytokines in pleural liquid for diagnosis of tuberculous pleurisy. Respir Med 2001;95:577-81. | Both index tests performed on <95% patients |
| 13. Aoe K, Hiraki A, Murakami T, Eda R, Maeda T, Sugi K, et al. Diagnostic significance of interferon-gamma in tuberculous pleural effusions. Chest 2003;123:740-4. | Only descriptive analysis |
| 14. Hiraki A, Aoe K, Eda R, Maeda T, Murakami T, Sugi K, et al. Comparison of six biological markers for the diagnosis of tuberculous pleuritis. Chest 2004;125:987-9. | Only descriptive analysis |

| | |
|---|---|
| 15. Tian RX, Gao ZC. Clinical investigation of the diagnostic value of interferon-gamma, interleukin-12 and adenosine deaminase isoenzyme in tuberculous pleurisy. Zhonghua Jie He He Hu Xi Za Zhi 2004;27:435-8. | Overlapping patient dataset |
| 16. Okamoto M, Hasegawa Y, Hara T, Hashimoto N, Imaizumi K, Shimokata K, et al. T-helper type 1/T-helper type 2 balance in malignant pleural effusions compared to tuberculous pleural effusions. Chest 2005;128:4030-5. | Only descriptive analysis |
| 17. Cok G, Parildar Z, Basol G, Kabaroglu C, Bayindir U, Habif S, et al. Pleural fluid neopterin levels in tuberculous pleurisy. Clin Biochem 2007;40:876-80. | Evaluating only one of the two index tests |
| 18. Fatolahzadeh B, Maleknejad P, Bahador A, Peeri-Dogaheh H, Alikhani MY, Radmanesh-Ahsani R. Evaluation of different primer sets for the rapid diagnosis of tuberculosis. Pak J Biol Sci 2007;10:107-11. | Not primarily focused on either index test |
| 19. Baba K, Sørnes S, Hoosen AA, Lekabe JM, Mpe MJ, Langeland N, et al. Evaluation of immune responses in HIV infected patients with pleural tuberculosis by the Quantiferon TB-Gold interferon-gamma assay. BMC Infect Dis 2008;8:35. | Evaluating only one of the two index tests |
| 20. Gerogianni I, Papala M, Tsopa P, Zigoulis P, Dimoulis A, Kostikas K, et al. Could IFN-gamma predict the development of residual pleural thickening in tuberculous pleurisy? Monaldi Arch Chest Dis 2008;69:18-23. | No data for non-tuberculous effusions |
| 21. Dheda K, Van-Zyl Smit RN, Sechi LA, Badri M, Meldau R, Symons G, et al. Clinical diagnostic utility of IP-10 and LAM antigen levels for the diagnosis of tuberculous pleural effusions in a high burden setting. PLoS One 2009;4:e4689. | Overlapping patient dataset |
| 22. Titarenko OT, Esmedliaeva DS, Perova TL, Alekseeva NP, D'Iakova M E, Popov M. Comparative significance of the biochemical markers of cell-mediated immunity in the diagnosis of tuberculous pleurisy. Klin Lab Diagn 2010;46-9. | Overlapping patient dataset |
| 23. Cirak AK, Komurcuoglu B, Tekgul S, Bilaceroglu S, Tasdogen N, Gunduz A. The diagnostic efficiency of QuantiferonTB®-Gold test in the diagnosis of tuberculous pleurisy. Int J Mycobacteriol 2012;1:180-4. | Evaluating only one of the two index tests |
| 24. Denkinger CM, Kalantri Y, Schumacher SG, Michael JS, Shankar D, Saxena A, et al. Challenges in the development of an immunochromatographic interferon-gamma test for diagnosis of pleural tuberculosis. PLoS One 2013;8:e85447. | Evaluating only one of the two index tests |
| 25. Liu F, Gao M, Zhang X, Du F, Jia H, Yang X, et al. Interferon-gamma release assay performance of pleural fluid and peripheral blood in pleural tuberculosis. PLoS One 2013;8:e83857. | Evaluating only one of the two index tests |
| 26. Marie MA, John J, Krishnappa LG, Gopalkrishnan S, Bindurani SR, Cs P. Role of interleukin-6, gamma interferon and adenosine deaminase markers in management of pleural effusion patients. West Indian Med J 2013;62:803-7. | Only descriptive analysis |
| 27. Liao M, Yang Q, Zhang J, Zhang M, Deng Q, Liu H, et al. Gamma interferon immunospot assay of pleural effusion mononuclear cells for diagnosis of tuberculous pleurisy. Clin Vaccine Immunol 2014;21:347-53. | Evaluating only one of the two index tests |
| 28. Liu F, Zhang X, Du F, Pan L, Liu Y, Jia H, et al. Comparison of interferon-gamma release assays and adenosine deaminase of pleural fluid for the diagnosis of pleural tuberculosis. Zhonghua Jie He He Hu Xi Za Zhi 2014;37:323-7. | Evaluating only one of the two index tests |
| 29. Meldau R, Peter J, Theron G, Calligaro G, Allwood B, Symons G, et al. Comparison of same day diagnostic tools including Gene Xpert and unstimulated IFN-gamma for the evaluation of pleural tuberculosis: a prospective cohort study. BMC Pulm Med 2014;14:58. | Both index tests performed on <95% patients |

| | |
|---|---|
| 30. Sánchez-Otero N, Rodríguez-Berrocal FJ, de la Cadena MP, Botana-Rial MI, Cordero OJ. Evaluation of pleural effusion sCD26 and DPP-IV as diagnostic biomarkers in lung disease. Sci Rep 2014;4:3999. | Evaluating only one of the two index tests |
| 31. Sun M, Yan D, Jiang S, Gu X, Ma W. Diagnostic value of interleukin-27 in tuberculous pleural effusion. Natl Med J China 2014;94:2641-4. | Not primarily focused on either index test |
| 32. Tang Y, Hua SC, Qin GX, Xu LJ, Jiang YF. Different subsets of macrophages in patients with new onset tuberculous pleural effusion. PLoS One 2014;9:e88343. | Evaluating only one of the two index tests |
| 33. Klimiuk J, Safianowska A, Chazan R, Korczyński P, Krenke R. Development and evaluation of the new predictive models in tuberculous pleuritis. Adv Exp Med Biol 2015;873:53-63. | Overlapping patient dataset |
| 34. Tang Y, Peng LP, Qin GX, Sun JT, Xu LJ, Jiang YF. CD4+CD25-Foxp3+ T cells play a role in tuberculous hydrothorax rather than malignant hydrothorax. J Transl Med 2015;13. | No data for non-tuberculous effusions |
| 35. Valdés L, San-José E, Ferreiro L, Golpe A, González-Barcala FJ, Toubes ME, et al. Predicting malignant and tuberculous pleural effusions through demographics and pleural fluid analysis of patients. Clin Resp J 2015;9:203-13. | Evaluating only one of the two index tests |
| 36. Adilistya T, Astrawinata DA, Nasir UZ. Use of pleural fluid interferon-gamma enzyme-linked immunospot assay in the diagnosis of pleural tuberculosis. Acta Med Indones 2016;48:41-7. | Evaluating only one of the two index tests |
| 37. Liu Y, Ou Q, Zheng J, Shen L, Zhang B, Weng X, et al. A combination of the Quantiferon-TB Gold In-Tube assay and the detection of adenosine deaminase improves the diagnosis of tuberculous pleural effusion. Emerg Microbes Infect 2016;5:e83. | Evaluating only one of the two index tests |
| 38. Teixeira LR, Dias MB, Sales RKB, Antonangelo L, Alvarenga VA, Puka J, et al. Profile ofmetalloproteinases and their association with inflammatory markers in pleural effusions. Lung 2016;194:1021-7. | Evaluating only one of the two index tests |
| 39. Bae MJ, Ryu S, Kim HJ, Cha SI, Kim CH, Lee J. Mycobacterium tuberculosis ESAT6 and CPF10 induce adenosine deaminase 2 mRNA expression in monocyte-derived macrophages. Tuberc Respir Dis (Seoul) 2017;80:77-82. | Not primarily focused on either index test |
| 40. Lin Y, Feng T, Lan J, Chen C, Qin Z, Wu Y, et al. Expression of toll-like receptor 2 and toll-like receptor 4 in tuberculous pleural effusion. Med Chem 2017;13:569-76. | Only descriptive analysis |
| 41. Ramadan SM, Laz NI, Eissa SAL, Elbatanouny MM, Mohammed MF. Diagnostic dilemma in tuberculous pleural effusion. Egyptian J Chest Dis Tuber 2017;66:327-30. | Evaluating only one of the two index tests |
| 42. Xu HY, Li CY, Su SS, Yang L, Ye M, Ye JR, et al. Diagnosis of tuberculous pleurisy with combination of adenosine deaminase and interferon-gamma immunospot assay in a tuberculosis-endemic population: A prospective cohort study. Medicine (Baltimore) 2017;96:e8412. | Evaluating only one of the two index tests |
| 43. Zhang Q, Zhou C. Comparison of laboratory testing methods for the diagnosis of tuberculous pleurisy in China. Sci Rep 2017;7:4549. | Evaluating only one of the two index tests |
| 44. Bayhan GI, Sayir F, Tanir G, Tuncer O. Pediatric pleural tuberculosis. Int J Mycobacteriology 2018;7:261-4. | No data for non-tuberculous effusions |

| | |
|---|---|
| 45. Kashyap B, Goyal N, Singh NP, Kaur IR. Diagnostic potential of circulating biomarkers in adenosine deaminase diagnosed pleural tuberculosis cases. Indian J Clin Biochem 2018;33:334-40. | Only descriptive analysis |
| 46. Korczyński P, Mierzejewski M, Krenke R, Safianowska A, Light RW. Cancer ratio and other new parameters for differentiation between malignant and nonmalignant pleural effusions. Polish Arch Intern Med 2018;128:354-61. | Overlapping patient dataset |
| 47. da Cunha Lisboa V, Ribeiro-Alves M, da Silva Correa R, Ramos Lopes I, Mafort TT, Santos AP, et al. Predominance of Th1 immune response in pleural effusion of patients with tuberculosis among other exudative etiologies. J Clin Microbiol 2019;58:e00927-19. | Only descriptive analysis |
| 48. Korczynski P, Klimiuk J, Safianowska A, Krenke R. Impact of age on the diagnostic yield of four different biomarkers of tuberculous pleural effusion. Tuberculosis 2019;114:24-9. | Overlapping patient dataset |
| 49. Meldau R, Randall P, Pooran A, Limberis J, Makambwa E, Dhansay M, et al. Same-day tools, including Xpert Ultra and IRISA-TB, for rapid diagnosis of pleural tuberculosis: a prospective observational study. J Clin Microbiol 2019;57:e00614-19. | Both index tests performed on <95% patients |
| 50. Qiu Y, Zeng W, Zhang H, Zhong X, Tang S, Zhang J. Comparison of pleural effusion features and biomarkers between talaromycosis and tuberculosis in non-human immunodeficiency virus-infected patients. BMC Infect Dis 2019;19:745. | Both index tests performed on <95% patients |
| 51. Tang Y, Zhang J, Huang H, He X, Zhang J, Ou M, et al. Pleural IFN-gamma release assay combined with biomarkers distinguished effectively tuberculosis from malignant pleural effusion. BMC Infect Dis 2019;19:55. | Evaluating only one of the two index tests |
| 52. Zhang M, Niu YR, Liu JY, Wei XS, Wang XR, Ye LL, et al. Interleukin-26 upregulates interleukin-22 production by human CD4+ T cells in tuberculous pleurisy. J Mol Med 2019;97:619-31. | Evaluating only one of the two index tests |
| 53. Jiang CG, Wang W, Zhou Q, Wu XZ, Wang XJ, Wang Z, et al. Influence of age on the diagnostic accuracy of soluble biomarkers for tuberculous pleural effusion: a post hoc analysis. BMC Pulm Med 2020;20:178. | Overlapping patient dataset |
| 54. Kim HJ, Ryu S, Choi SH, Seo H, Yoo SS, Lee SY, et al. Comparison of biochemical parameters and chemokine levels in pleural fluid between patients with anergic and non-anergic tuberculous pleural effusion. Tuberculosis 2020;123. | Evaluating only one of the two index tests |
| 55. Radhakrishnan P, Mathanraj S. Role of pleural fluid C-reactive protein in the aetiological diagnosis of exudative pleural effusion. J Clin Diag Res 2020;14:OC04-OC7. | Evaluating only one of the two index tests |
| 56. Zhang BY, Yu ZM, Yang QL, Liu QQ, Chen HX, Wu J, et al. Serial anti-tuberculous immune responses during the follow-up of patients with tuberculous pleurisy. Medicine (United States) 2020;99. | No data for non-tuberculous effusions |
| 57. Zhang T, Wan B, Wang L, Li C, Xu Y, Wang X, et al. The diagnostic yield of closed needle pleural biopsy in exudative pleural effusion: a retrospective 10-year study. Ann Transl Med 2020;8:491. | Evaluating only one of the two index tests |
