# Supplementary material for: Comparative accuracy of pleural fluid unstimulated interferon-gamma and adenosine deaminase for diagnosing pleural tuberculosis: A systematic review and meta-analysis
Source: PLoS One. 2021 Jun 24;16(6):e0253525. doi: 10.1371/journal.pone.0253525 (PMC8224977; doi:10.1371/journal.pone.0253525)
Supplement: S2 Table — (PDF) [file pone.0253525.s002.pdf]

S2 Table. Pleural fluid assays for the index tests, and their results, from the studies included in data synthesis.

Primary author, publication year	Pleural fluid adenosine deaminase							Pleural fluid unstimulated interferon-gamma						
	TPE / other effusions	Assay technique	Assay threshold	TP	FN	TN	FP	TPE / other effusions	Assay technique	Assay threshold	TP	FN	TN	FP
Hsu, 1989	19 / 29	Guisti	50 IU/L	18	1	21	8	19 / 29	ELISA	10 U/L	18	1	26	3
Ribera, 1990	48 / 114	Guisti	43 IU/L	48	0	105	9	48 / 114	RIA	2 U/mL	48	0	114	0
Aoki, 1994	11 / 28	Non-Guisti	45 IU/L	9	2	25	3	11 / 28	ELISA	0.3 U/mL	11	0	28	0
Jeon, 1998	20 / 20	Guisti	45 IU/L	17	3	18	2	20 / 20	ELISA	100 pg/mL	18	2	17	3
Kim, 1998	40 / 42	Guisti	50 IU/L<br>70 IU/L	35<br>29	5<br>11	41<br>42	1<br>0	40 / 42	ELISA	140 pg/mL	39	1	42	0
Zhu, 1999	25 / 33	Guisti	40 IU/L	22	3	30	3	25 / 33	ELISA	140 pg/mL	24	1	32	1
Villegas, 2000	61 / 70	Guisti	45 IU/L	53	8	60	10	58 / 70	ELISA	6 U/mL	45	13	68	2
Poyraz, 2004	15 / 30	Guisti	45 IU/L	13	2	30	0	15 / 30	ELISA	12 pg/mL	13	2	29	1
El-Ansary, 2005	15 / 24	Guisti	35 IU/L	12	3	22	2	15 / 24	ELISA	3.1 U/mL	14	1	24	0
Gao, 2005	141 / 49	Non-Guisti	40.3 IU/L	116	25	43	6	141 / 49	ELISA	61.7 pg/mL	119	22	47	2
Okamoto, 2005	11 / 32	Non-Guisti	32 IU/L	11	0	31	1	11 / 32	ELISA	99.3 pg/mL	10	1	31	1
Park, 2005	39 / 25	NS	40 IU/L	38	1	19	6	39 / 26	ELISA	300 pg/mL	38	1	25	1
Sharma, 2005	35 / 17	Guisti	33 IU/L	32	3	17	0	35 / 17	ELISA	176.5 pg/mL	34	1	17	0
Morimoto, 2006	19 / 46	Non-Guisti	50 IU/L<br>57 IU/L<br>58 IU/L	15<br>15<br>14	4<br>4<br>5	44<br>45<br>45	2<br>1<br>1	19 / 46	ELISA	195 pg/mL<br>248 pg/mL<br>260 pg/mL	16<br>16<br>15	3<br>3<br>4	42<br>43<br>43	4<br>3<br>3
Ariga, 2007	27 / 47	NS	40.7 IU/L	22	5	43	4	28 / 47	ELISA	2.456 U/mL	24	4	45	2
Daniil, 2007	12 / 60	Guisti	42.2 IU/L	9	3	47	13	12 / 60	NS	NS	9	3	47	13

Xue, 2007	45 / 42	Non-Guisti	40 U/L	36	9	37	5	45 / 42	ELISA	95.3 ng/L	39	6	40	2
Krenke, 2008	28 / 66	Guisti	40.3 U/L	28	0	62	4	28 / 66	ELISA	75 pg/mL	28	0	65	1
Titarenko, 2008	35 / 53	Guisti	35 U/L	34	1	52	1	35 / 53	ELISA	180 pg/mL	33	2	51	2
Dheda, 2009	55 / 19	Guisti	30 U/L	52	3	13	6	55 / 19	ELISA	0.3 U/mL	54	1	19	0
Valdes, 2009	39 / 57	Guisti	54.3 U/L	38	1	53	4	39 / 57	ELISA	169 pg/mL	32	7	53	4
Wu, 2010	23 / 56	Non-Guisti	19.5 U/L	22	1	45	11	23 / 56	ELISA	42.95 pg/mL	20	3	53	3
Ambade, 2011	48 / 33	Guisti	71 IU/L	38	10	25	8	48 / 33	ELISA	1090 pg/mL	42	6	28	5
Kalantri, 2011	154 / 50	Guisti	44.75 IU/L	122	32	46	4	154 / 50	ELISA	NS	129	25	48	2
Liu, 2011	24 / 42	Guisti	30 IU/L	17	7	40	2	24 / 42	Bead array	70 pg/mL	22	2	41	1
Wang, 2012	78 / 44	Guisti	40 IU/L	73	5	40	4	78 / 44	ELISA	225 ng/L	71	7	39	5
Keng, 2013	31 / 57	Guisti	40 IU/L<br>15.5 IU/L	14<br>26	17<br>5	56<br>50	1<br>7	31 / 57	ELISA	45 pg/mL<br>75 pg/mL	24<br>22	7<br>9	55<br>56	2<br>1
Khan, 2013	72 / 31	Guisti	16.65 IU/L	62	10	23	8	72 / 31	ELISA	0.5 pg/mL	72	0	31	0
Lee, 2013	60 / 160	Guisti	28 IU/L	53	7	147	13	60 / 160	ELISA	0.89 U/L	55	5	157	3
Wu, 2013	40 / 41	Guisti	24.5 IU/L	35	5	35	6	40 / 41	ELISA	355.2 ng/L	36	4	40	1
Li, 2014	47 / 43	Non-Guisti	39 U/L	36	11	36	7	47 / 43	ELISA	103.65 ng/L	38	9	35	8
Valdes, 2014	70 / 361	Non-Guisti	56 U/L	69	1	337	24	70 / 361	ELISA	108.2 pg/mL	63	7	327	34
Yurt, 2014	43 / 50	Guisti	40.68 U/L	38	5	44	6	43 / 50	ELISA	110 U/L	32	11	34	16
Ali, 2015	20 / 20	Guisti	35.5 IU/L	17	3	17	3	20 / 20	ELISA	0.5 U/mL	18	2	19	1
Dong, 2015	63 / 50	Guisti	45 IU/L	45	18	47	3	63 / 50	ELISA	138.5 pg/mL	59	4	41	9
Klimiuk, 2015	44 / 159	NS	40 IU/L	39	5	148	11	44 / 159	ELISA	118.7 pg/mL	43	1	157	2
Shu, 2015	35 / 60	Guisti	40 IU/L	14	21	59	1	35 / 60	ELISA	75 pg/mL	23	12	58	2

Jethani, 2016	45 / 45	Guisti	40 IU/L	40	5	45	0	45 / 45	ELISA	200 pg/mL	44	1	44	1
Chung, 2017	106 / 230	Non-Guisti	46.1 IU/L	93	13	217	13	106 / 230	ELISA	38.3 pg/mL	100	6	220	10
Santos, 2018	33 / 46	Guisti	25.8 IU/L	32	1	39	7	33 / 46	ELISA	2.33 U/mL	29	4	45	1
Wang, 2018 (a)	51 / 103	Non-Guisti	21.4 IU/L	45	6	89	14	51 / 103	ELISA	116.1 ng/L	47	4	98	5
(b)	44 / 76	Non-Guisti	21.4 IU/L	39	5	66	10	44 / 76	ELISA	116.1 ng/L	41	3	74	2
Faria, 2019	15 / 105	Guisti	29.6 IU/L	13	2	98	7	15 / 105	Magnetic	1.8 pg/mL	14	1	98	7
Li, 2019	42 / 38	Non-Guisti	18.6 IU/L	33	9	35	3	42 / 38	ELISA	54.4 ng/L	27	17	34	4
Zhang, 2020	108 / 134	Non-Guisti	19.81 IU/L	94	14	121	13	108 / 134	Bead array	2.45 pg/mL	99	9	123	11

ELISA Enzyme-linked immunosorbent assay, FN False negative, FP False positive, NS Not specified, RIA Radioimmunoassay, TN True negative, TP True positive, TPE Tuberculous pleural effusion
