# Supplementary material for: Comparative accuracy of pleural fluid unstimulated interferon-gamma and adenosine deaminase for diagnosing pleural tuberculosis: A systematic review and meta-analysis
Source: PLoS One. 2021 Jun 24;16(6):e0253525. doi: 10.1371/journal.pone.0253525 (PMC8224977; doi:10.1371/journal.pone.0253525)
Supplement: S3 Table — (PDF) [file pone.0253525.s003.pdf]

S3 Table. Diagnostic accuracy estimates for pleural fluid unstimulated interferon-gamma (IFN-γ) and adenosine deaminase (ADA) assays.

Author, year	Test	Sensitivity	Specificity	Positive likelihood ratio	Negative likelihood ratio	Diagnostic odds ratio
Hsu, 1989	IFN-γ	0.95 (0.74-1.00)	0.90 (0.73-0.98)	9.16 (3.12-26.88)	0.06 (0.01-0.40)	156.00 (15.00-1622.07)
	ADA	0.95 (0.74-1.00)	0.72 (0.53-0.87)	3.43 (1.89-6.25)	0.07 (0.01-0.50)	47.25 (5.38-414.70)
Ribera, 1990	IFN-γ	1.00 (0.93-1.00)	1.00 (0.97-1.00)	113.68 (16.14-800.54)	0.02 (0.00-0.14)	5635.00 (345.45-91918)
	ADA	1.00 (0.93-1.00)	0.92 (0.86-0.96)	11.37 (6.28-20.59)	0.02 (0.00-0.15)	519.40 (64.67-4171.37)
Aoki, 1994	IFN-γ	1.00 (0.72-1.00)	1.00 (0.88-1.00)	27.69 (4.01-191.44)	0.08 (0.01-0.52)	348.00 (20.08-6029.88)
	ADA	0.82 (0.48-0.98)	0.89 (0.72-0.98)	7.64 (2.53-23.05)	0.20 (0.06-0.72)	37.50 (5.36-262.17)
Jeon, 1998	IFN-γ	0.90 (0.68-0.99)	0.85 (0.62-0.97)	6.00 (2.09-17.21)	0.12 (0.03-0.44)	51.00 (7.57-343.73)
	ADA	0.85 (0.62-0.97)	0.90 (0.68-0.99)	8.50 (2.25-32.06)	0.17 (0.06-0.48)	51.00 (7.57-343.73)
Kim, 1998	IFN-γ	0.98 (0.87-1.00)	1.00 (0.92-1.00)	41.90 (6.03-291.24)	0.05 (0.01-0.19)	860.00 (75.05-9854.61)
	ADA	0.88 (0.73-0.96)	0.98 (0.87-1.00)	36.75 (5.28-255.74)	0.13 (0.06-0.29)	287.00 (32.00-2574.43)
Zhu, 1999	IFN-γ	0.96 (0.80-1.00)	0.97 (0.84-1.00)	31.68 (4.59-218.63)	0.04 (0.01-0.28)	768.00 (45.69-12908.9)
	ADA	0.88 (0.69-0.97)	0.91 (0.76-0.98)	9.68 (3.26-28.75)	0.13 (0.05-0.38)	73.33 (13.50-398.31)
Villegas, 2000	IFN-γ	0.78 (0.65-0.87)	0.97 (0.90-1.00)	27.16 (6.88-107.18)	0.23 (0.14-0.37)	117.69 (25.34-546.58)
	ADA	0.87 (0.76-0.94)	0.86 (0.75-0.93)	6.08 (3.40-10.88)	0.15 (0.08-0.29)	39.75 (14.62-108.10)
Poyraz, 2004	IFN-γ	0.87 (0.60-0.98)	0.97 (0.83-1.00)	26.00 (3.75-180.42)	0.14 (0.04-0.50)	188.50 (15.66-2269.01)
	ADA	0.87 (0.60-0.98)	1.00 (0.88-1.00)	26.35 (3.78-183.68)	0.18 (0.07-0.51)	144.67 (13.80-1516.19)
El-Ansary, 2005	IFN-γ	0.93 (0.68-1.00)	1.00 (0.86-1.00)	22.94 (3.33-158.01)	0.12 (0.03-0.45)	187.50 (15.63-2248.72)
	ADA	0.80 (0.52-0.96)	0.92 (0.73-0.99)	9.60 (2.49-37.06)	0.22 (0.08-0.60)	44.00 (6.43-300.87)
Gao, 2005	IFN-γ	0.84 (0.77-0.90)	0.96 (0.86-1.00)	20.68 (5.31-80.50)	0.16 (0.11-0.24)	127.11 (28.75-561.98)
	ADA	0.82 (0.75-0.88)	0.88 (0.75-0.95)	6.72 (3.16-14.27)	0.20 (0.14-0.29)	33.25 (12.77-86.61)
Okamoto, 2005	IFN-γ	0.91 (0.59-1.00)	0.97 (0.84-1.00)	29.09 (4.19-202.06)	0.09 (0.01-0.61)	310.00 (17.72-5423.85)
	ADA	1.00 (0.72-1.00)	0.97 (0.84-1.00)	15.69 (4.05-60.75)	0.08 (0.01-0.54)	192.00 (15.91-2316.81)

Park, 2005	IFN-γ	0.97 (0.87-1.00)	0.96 (0.80-1.00)	25.33 (3.70-173.24)	0.03 (0.00-0.18)	950.00 (56.77-15896.4)
	ADA	0.97 (0.87-1.00)	0.76 (0.55-0.91)	4.06 (2.02-8.17)	0.03 (0.00-0.24)	120.33 (13.50-1072.47)
Sharma, 2005	IFN-γ	0.97 (0.85-1.00)	1.00 (0.80-1.00)	17.97 (2.66-121.28)	0.06 (0.01-0.22)	315.00 (26.73-3712.37)
	ADA	0.91 (0.77-0.98)	1.00 (0.80-1.00)	16.95 (2.51-114.55)	0.11 (0.04-0.29)	148.50 (15.41-1430.67)
Morimoto, 2006	IFN-γ	0.84 (0.60-0.97)	0.93 (0.82-0.99)	12.91 (4.25-39.23)	0.17 (0.06-0.48)	76.44 (13.96-418.50)
	ADA	0.79 (0.54-0.94)	0.98 (0.88-1.00)	36.32 (5.15-255.87)	0.22 (0.09-0.51)	168.75 (17.47-1629.94)
Ariga, 2007	IFN-γ	0.86 (0.67-0.96)	0.96 (0.85-0.99)	20.14 (5.15-78.83)	0.15 (0.06-0.37)	135.00 (23.04-791.14)
	ADA	0.81 (0.62-0.94)	0.91 (0.80-0.98)	9.57 (3.69-24.87)	0.20 (0.09-0.45)	47.30 (11.53-194.05)
Daniil, 2007	IFN-γ	0.75 (0.43-0.95)	0.78 (0.66-0.88)	3.46 (1.94-6.19)	0.32 (0.12-0.86)	10.85 (2.56-45.95)
	ADA	0.75 (0.43-0.95)	0.78 (0.66-0.88)	3.46 (1.94-6.19)	0.32 (0.12-0.86)	10.85 (2.56-45.95)
Xue, 2007	IFN-γ	0.87 (0.73-0.95)	0.95 (0.84-0.99)	18.20 (4.68-70.72)	0.14 (0.07-0.30)	130.00 (24.72-683.69)
	ADA	0.80 (0.65-0.90)	0.88 (0.74-0.96)	6.72 (2.91-15.50)	0.23 (0.13-0.41)	29.60 (9.04-96.87)
Krenke, 2008	IFN-γ	1.00 (0.88-1.00)	0.98 (0.92-1.00)	32.87 (8.38-128.95)	0.03 (0.00-0.24)	957.00 (83.43-10978.0)
	ADA	1.00 (0.88-1.00)	0.94 (0.85-0.98)	13.15 (5.64-30.64)	0.04 (0.01-0.25)	365.40 (40.83-3270.21)
Titarenko, 2008	IFN-γ	0.94 (0.81-0.99)	0.96 (0.87-1.00)	24.99 (6.40-97.54)	0.06 (0.02-0.23)	420.75 (56.47-3134.79)
	ADA	0.97 (0.85-1.00)	0.98 (0.90-1.00)	51.49 (7.38-359.07)	0.03 (0.00-0.20)	1768.00 (106.94-29229)
Dheda, 2009	IFN-γ	0.98 (0.90-1.00)	1.00 (0.82-1.00)	20.26 (2.99-137.30)	0.04 (0.01-0.14)	550.00 (47.25-6401.73)
	ADA	0.95 (0.85-0.99)	0.68 (0.43-0.87)	2.99 (1.54-5.82)	0.08 (0.03-0.25)	37.56 (8.27-170.57)
Valdes, 2009	IFN-γ	0.82 (0.66-0.92)	0.93 (0.83-0.98)	11.69 (4.49-30.42)	0.19 (0.10-0.38)	60.57 (16.43-223.25)
	ADA	0.97 (0.87-1.00)	0.93 (0.83-0.98)	13.88 (5.39-35.77)	0.03 (0.00-0.19)	503.50 (54.11-4685.10)
Wu, 2010	IFN-γ	0.87 (0.66-0.97)	0.95 (0.85-0.99)	16.23 (5.34-49.36)	0.14 (0.05-0.40)	117.78 (21.93-632.53)
	ADA	0.96 (0.78-1.00)	0.80 (0.68-0.90)	4.87 (2.85-8.33)	0.05 (0.01-0.37)	90.00 (10.92-742.09)
Ambade, 2011	IFN-γ	0.88 (0.75-0.95)	0.85 (0.68-0.95)	5.78 (2.56-13.04)	0.15 (0.07-0.32)	39.20 (10.90-140.92)
	ADA	0.79 (0.65-0.90)	0.76 (0.58-0.89)	3.27 (1.76-6.07)	0.28 (0.15-0.49)	11.88 (4.12-34.20)

Kalantri, 2011	IFN-γ	0.84 (0.77-0.89)	0.96 (0.86-1.00)	20.94 (5.38-81.57)	0.17 (0.12-0.24)	123.84 (28.25-542.88)
	ADA	0.79 (0.72-0.85)	0.92 (0.81-0.98)	9.90 (3.85-25.44)	0.23 (0.16-0.31)	43.84 (14.69-130.84)
Liu, 2011	IFN-γ	0.92 (0.73-0.99)	0.98 (0.87-1.00)	38.50 (5.53-267.98)	0.09 (0.02-0.32)	451.00 (38.70-5256.25)
	ADA	0.71 (0.49-0.87)	0.95 (0.84-0.99)	14.88 (3.75-58.93)	0.31 (0.16-0.57)	48.57 (9.14-258.22)
Wang, 2012	IFN-γ	0.91 (0.82-0.96)	0.89 (0.75-0.96)	8.01 (3.50-18.34)	0.10 (0.05-0.21)	79.11 (23.54-265.92)
	ADA	0.94 (0.86-0.98)	0.91 (0.78-0.97)	10.29 (4.04-26.25)	0.07 (0.03-0.17)	146.00 (37.09-574.65)
Keng, 2013	IFN-γ	0.77 (0.59-0.90)	0.96 (0.88-1.00)	22.06 (5.58-87.23)	0.23 (0.12-0.45)	94.29 (18.23-487.51)
	ADA	0.84 (0.66-0.95)	0.88 (0.76-0.95)	6.83 (3.36-13.90)	0.18 (0.08-0.41)	37.14 (10.73-128.56)
Khan, 2013	IFN-γ	1.00 (0.95-1.00)	1.00 (0.89-1.00)	32.55 (4.72-224.33)	0.01 (0.00-0.10)	2336.00 (141.66-38522)
	ADA	0.86 (0.76-0.93)	0.74 (0.55-0.88)	3.34 (1.82-6.10)	0.19 (0.10-0.35)	17.83 (6.27-50.71)
Lee, 2013	IFN-γ	0.92 (0.82-0.97)	0.98 (0.95-1.00)	48.89 (15.90-150.37)	0.08 (0.04-0.20)	575.67 (133.17-2488.6)
	ADA	0.88 (0.77-0.95)	0.92 (0.87-0.96)	10.87 (6.40-18.45)	0.13 (0.06-0.26)	85.62 (32.42-226.08)
Wu, 2013	IFN-γ	0.90 (0.76-0.97)	0.98 (0.87-1.00)	36.90 (5.31-256.44)	0.10 (0.04-0.26)	360.00 (38.44-3371.77)
	ADA	0.88 (0.73-0.96)	0.85 (0.71-0.94)	5.98 (2.83-12.64)	0.15 (0.06-0.34)	40.83 (11.40-146.27)
Li, 2014	IFN-γ	0.81 (0.67-0.91)	0.81 (0.67-0.92)	4.35 (2.29-8.25)	0.24 (0.13-0.43)	18.47 (6.42-53.17)
	ADA	0.77 (0.62-0.88)	0.84 (0.69-0.93)	4.71 (2.35-9.44)	0.28 (0.16-0.48)	16.83 (5.86-48.30)
Valdes, 2014	IFN-γ	0.90 (0.80-0.96)	0.91 (0.87-0.93)	9.56 (6.87-13.28)	0.11 (0.05-0.22)	86.56 (36.74-203.94)
	ADA	0.99 (0.92-1.00)	0.93 (0.90-0.96)	14.83 (10.06-21.85)	0.02 (0.00-0.11)	968.88 (128.90-7282.4)
Yurt, 2014	IFN-γ	0.74 (0.59-0.86)	0.68 (0.53-0.80)	2.33 (1.50-3.61)	0.38 (0.22-0.65)	6.18 (2.50-15.31)
	ADA	0.88 (0.75-0.96)	0.88 (0.76-0.95)	7.36 (3.45-15.72)	0.13 (0.06-0.30)	55.73 (15.75-197.21)
Ali, 2015	IFN-γ	0.90 (0.68-0.99)	0.95 (0.75-1.00)	18.00 (2.65-122.28)	0.11 (0.03-0.39)	171.00 (14.24-2053.25)
	ADA	0.85 (0.62-0.97)	0.85 (0.62-0.97)	5.67 (1.96-16.35)	0.18 (0.06-0.51)	32.11 (5.66-182.18)
Dong, 2015	IFN-γ	0.94 (0.85-0.98)	0.82 (0.69-0.91)	5.20 (2.87-9.43)	0.08 (0.03-0.20)	67.19 (19.38-232.98)
	ADA	0.71 (0.59-0.82)	0.94 (0.83-0.99)	11.90 (3.93-36.06)	0.30 (0.20-0.45)	39.17 (10.79-142.11)

Klimiuk, 2015	IFN-γ	0.98 (0.88-1.00)	0.99 (0.96-1.00)	77.69 (19.59-308.17)	0.02 (0.00-0.16)	3375.50 (298.94-38114)
	ADA	0.89 (0.75-0.96)	0.93 (0.88-0.97)	12.81 (7.17-22.88)	0.12 (0.05-0.28)	104.95 (34.43-319.86)
Shu, 2015	IFN-γ	0.66 (0.48-0.81)	0.97 (0.88-1.00)	19.71 (4.94-78.63)	0.35 (0.22-0.56)	55.58 (11.53-267.95)
	ADA	0.40 (0.24-0.58)	0.98 (0.91-1.00)	24.00 (3.30-174.78)	0.61 (0.46-0.80)	39.33 (4.87-317.69)
Jethani, 2016	IFN-γ	0.98 (0.88-1.00)	0.98 (0.88-1.00)	44.00 (6.33-305.75)	0.02 (0.00-0.16)	1936.00 (117.36-31936)
	ADA	0.89 (0.76-0.96)	1.00 (0.92-1.00)	41.00 (5.88-285.90)	0.13 (0.06-0.28)	314.33 (36.31-2721.41)
Chung, 2017	IFN-γ	0.94 (0.88-0.98)	0.96 (0.92-0.98)	21.70 (11.81-39.85)	0.06 (0.03-0.13)	366.67 (129.68-1036.7)
	ADA	0.88 (0.80-0.93)	0.94 (0.91-0.97)	15.52 (9.11-26.45)	0.13 (0.08-0.22)	119.41 (53.32-267.42)
Santos, 2018	IFN-γ	0.88 (0.72-0.97)	0.98 (0.88-1.00)	40.42 (5.79-282.06)	0.12 (0.05-0.31)	326.25 (34.72-3065.98)
	ADA	0.97 (0.84-1.00)	0.85 (0.71-0.94)	6.37 (3.21-12.64)	0.04 (0.01-0.25)	178.29 (20.83-1525.68)
Wang, 2018 (a)	IFN-γ	0.92 (0.81-0.98)	0.95 (0.89-0.98)	18.98 (8.04-44.81)	0.08 (0.03-0.21)	230.30 (59.11-897.29)
	ADA	0.88 (0.76-0.96)	0.86 (0.78-0.92)	6.49 (3.95-10.67)	0.14 (0.06-0.29)	47.68 (17.17-132.40)
Wang, 2018 (b)	IFN-γ	0.93 (0.81-0.99)	0.97 (0.91-1.00)	35.41 (9.00-139.33)	0.07 (0.02-0.21)	505.67 (81.16-3150.48)
	ADA	0.89 (0.75-0.96)	0.87 (0.77-0.94)	6.74 (3.74-12.12)	0.13 (0.06-0.30)	51.48 (16.40-161.64)
Faria, 2019	IFN-γ	0.93 (0.68-1.00)	0.93 (0.87-0.97)	14.00 (6.76-29.00)	0.07 (0.01-0.47)	196.00 (22.40-1714.62)
	ADA	0.87 (0.60-0.98)	0.93 (0.87-0.97)	13.00 (6.19-27.32)	0.14 (0.04-0.52)	91.00 (17.05-485.62)
Li, 2019	IFN-γ	0.61 (0.46-0.76)	0.89 (0.75-0.97)	5.83 (2.24-15.17)	0.43 (0.29-0.64)	13.50 (4.06-44.85)
	ADA	0.79 (0.63-0.90)	0.92 (0.79-0.98)	9.95 (3.32-29.82)	0.23 (0.13-0.42)	42.78 (10.65-171.83)
Zhang, 2020	IFN-γ	0.92 (0.85-0.96)	0.92 (0.86-0.96)	11.17 (6.32-19.73)	0.09 (0.05-0.17)	123.00 (49.03-308.59)
	ADA	0.87 (0.79-0.93)	0.90 (0.84-0.95)	8.97 (5.32-15.12)	0.14 (0.09-0.23)	62.49 (28.04-139.30)

Figures in parentheses are 95% confidence intervals
