# Supplementary material for: Comparative accuracy of pleural fluid unstimulated interferon-gamma and adenosine deaminase for diagnosing pleural tuberculosis: A systematic review and meta-analysis
Source: PLoS One. 2021 Jun 24;16(6):e0253525. doi: 10.1371/journal.pone.0253525 (PMC8224977; doi:10.1371/journal.pone.0253525)
Supplement: S4 Table — (PDF) [file pone.0253525.s004.pdf]

S4 Table. Evaluation of factors affecting individual summary diagnostic accuracy estimates of pleural fluid assays.

Factor	Pleural fluid adenosine deaminase			Pleural fluid unstimulated interferon-gamma		
	**Group (number of studies)**	**Sensitivity**	**Specificity**	**Group (number of studies)**	**Sensitivity**	**Specificity**
Overall	- Total (N=45)	0.88 (0.85-0.91)	0.91 (0.89-0.92)	- Total (N=45)	0.91 (0.89-0.94)	0.96 (0.94-0.97)
Year of publication	- Up to 2010 (N=22)<br>- After 2010 (N=23)	0.91 (0.87-0.94)<br>0.85 (0.81-0.89)	0.91 (0.87-0.94)<br>0.91 (0.89-0.93)	- Up to 2010 (N=22)<br>- After 2010 (N=23)	0.92 (0.88-0.95)<br>0.90 (0.86-0.93)	0.97 (0.95-0.98)<br>0.95 (0.92-0.96)
Design of study	- Prospective (N=43)<br>- Not prospective (N=2)	0.87 (0.84-0.90)<br>Not estimable	0.91 (0.89-0.93)<br>Not estimable	- Prospective (N=43)<br>- Not prospective (N=2)	0.91 (0.88-0.93)<br>Not estimable	0.95 (0.94-0.97)<br>Not estimable
Burden of TB in country of study	- Not high (N=21)<br>- High (N=24)	0.89 (0.83-0.93)<br>0.87 (0.84-0.90)	0.92 (0.89-0.94)<br>0.90 (0.87-0.92)	- Not high (N=21)<br>- High (N=24)	0.91 (0.86-0.94)<br>0.92 (0.88-0.95)	0.97 (0.93-0.98)<br>0.95 (0.93-0.96)
Proportion of TB patients in study population	- >50% (N=12)<br>- <=50% (N=33)	0.85 (0.80-0.89)<br>0.89 (0.86-0.92)	0.86 (0.81-0.90)<br>0.92 (0.90-0.93)	- >50% (N=12)<br>- <=50% (N=33)	0.92 (0.84-0.96)<br>0.91 (0.88-0.94)	0.94 (0.88-0.97)<br>0.96 (0.94-0.97)
Effusion characteristics	- Only exudates (N=22)<br>- Transudates also (N=23)	0.88 (0.84-0.91)<br>0.88 (0.83-0.92)	0.90 (0.86-0.92)<br>0.91 (0.89-0.93)	- Only exudates (N=22)<br>- Transudates also (N=23)	0.93 (0.90-0.95)<br>0.89 (0.84-0.93)	0.96 (0.93-0.97)<br>0.96 (0.93-0.97)
Assay methodology	- Guisti technique (N=29)<br>- Other, or not stated (N=16)	0.88 (0.84-0.92)<br>0.88 (0.83-0.91)	0.91 (0.88-0.93)<br>0.90 (0.88-0.92)	- ELISA (N=40)<br>- Other, or not stated (N=5)	0.91 (0.88-0.93)<br>0.94 (0.81-0.98)	0.96 (0.94-0.97)<br>0.95 (0.84-0.99)
Reference standard	- Definite (N=24)<br>- Composite (N=21)	0.89 (0.84-0.93)<br>0.87 (0.84-0.90)	0.92 (0.89-0.94)<br>0.89 (0.86-0.92)	- Definite (N=24)<br>- Composite (N=21)	0.92 (0.88-0.94)<br>0.91 (0.86-0.94)	0.96 (0.92-0.97)<br>0.96 (0.94-0.97)
Blinding in study	- Yes (N=5)<br>- No, or not stated (N=40)	0.89 (0.80-0.94)<br>0.88 (0.85-0.91)	0.88 (0.80-0.93)<br>0.91 (0.89-0.93)	- Yes (N=5)<br>- No, or not stated (N=40)	0.92 (0.85-0.96)<br>0.91 (0.88-0.94)	0.96 (0.91-0.98)<br>0.96 (0.94-0.97)

Figures in parentheses are 95% confidence intervals for the summary sensitivity and specificity estimates
ELISA Enzyme-linked immunosorbent assay, TB Tuberculosis
