# Supplementary material for: Comparative accuracy of pleural fluid unstimulated interferon-gamma and adenosine deaminase for diagnosing pleural tuberculosis: A systematic review and meta-analysis
Source: PLoS One. 2021 Jun 24;16(6):e0253525. doi: 10.1371/journal.pone.0253525 (PMC8224977; doi:10.1371/journal.pone.0253525)
Supplement: S5 Table — (PDF) [file pone.0253525.s005.pdf]

S5 Table. Parameters and summary estimates from hierarchical summary receiver operating characteristic (HSROC) models evaluating diagnostic accuracy of pleural fluid adenosine deaminase (ADA) and unstimulated interferon-gamma (IFN-γ).

Index tests, parameters, and summary estimates	Models for a single index test		Models including type of test (ADA or IFN-γ) as covariate		
	IFN-γ	ADA	Covariate effect assessed on accuracy, threshold, and shape	Covariate effect assessed on accuracy and threshold only	Covariate effect assessed on accuracy alone
Accuracy parameter (alpha)					
- IFN-γ	5.43 (4.85-6.01)		5.03 (4.65-5.41)	5.13 (4.70-5.55)	5.06 (4.68-5.44)
- ADA		4.35 (3.96-4.74)	4.44 (3.96-4.92)	4.31 (3.93-4.70)	4.27 (3.90-4.64)
Threshold parameter (theta)					
- IFN-γ	-0.22 (-0.72-0.29)		-0.10 (-0.81-0.62)	-0.47 (-1.01-0.07)	-0.19 (-0.62-0.24)
- ADA		-0.41 (-0.99-0.17)	-0.57 (-1.14-0.00)	-0.33 (-0.79-0.13)	-0.19 (-0.62-0.24)
Shape parameter (beta)					
- IFN-γ	0.11 (-0.30-0.52)		0.16 (-0.44-0.77)	-0.17 (-0.59-0.26)	0.02 (-0.36-0.39)
- ADA		-0.25 (-0.79-0.29)	-0.40 (-0.92-0.12)	-0.17 (-0.59-0.26)	0.02 (-0.36-0.39)
Summary sensitivity					
- IFN-γ	0.91 (0.89-0.94)		0.90 (0.88-0.92)	0.91 (0.88-0.93)	0.91 (0.89-0.93)
- ADA		0.88 (0.85-0.91)	0.88 (0.85-0.91)	0.88 (0.85-0.90)	0.87 (0.85-0.90)
Summary specificity					
- IFN-γ	0.96 (0.94-0.97)		0.94 (0.93-0.96)	0.94 (0.93-0.95)	0.94 (0.92-0.95)
- ADA		0.91 (0.89-0.92)	0.91 (0.89-0.92)	0.91 (0.89-0.92)	0.91 (0.89-0.93)
Model fit					
- -2 log likelihood	409.6	467.8	909.0	911.3	914.2
Relative accuracy (IFN-γ *vs*. ADA)					
- Relative sensitivity			1.02 (1.00-1.05)	1.03 (1.01-1.05)	1.04 (1.02-1.06)
- Difference in sensitivity			0.02 (-0.00-0.05)	0.03 (0.00-0.05)	0.04 (0.02-0.06)
- Relative specificity			1.04 (1.02-1.06)	1.04 (1.02-1.05)	1.03 (1.02-1.04)
- Difference in specificity			0.04 (0.02-0.05)	0.03 (0.01-0.05)	0.03 (0.01-0.04)
- Relative diagnostic odds ratio (RDOR)			2.13 (1.59-2.85)	2.22 (1.68-2.94)	2.21 (1.66-2.93)

Figures in parentheses are 95% confidence intervals; See Fig E4 for the corresponding HSROC model plots.
