# Supplementary figures and images for: Comparative accuracy of pleural fluid unstimulated interferon-gamma and adenosine deaminase for diagnosing pleural tuberculosis: A systematic review and meta-analysis
Source: PLoS One. 2021 Jun 24;16(6):e0253525. doi: 10.1371/journal.pone.0253525 (PMC8224977; doi:10.1371/journal.pone.0253525)

**S1 Fig.** Risk of bias and applicability concerns summary.

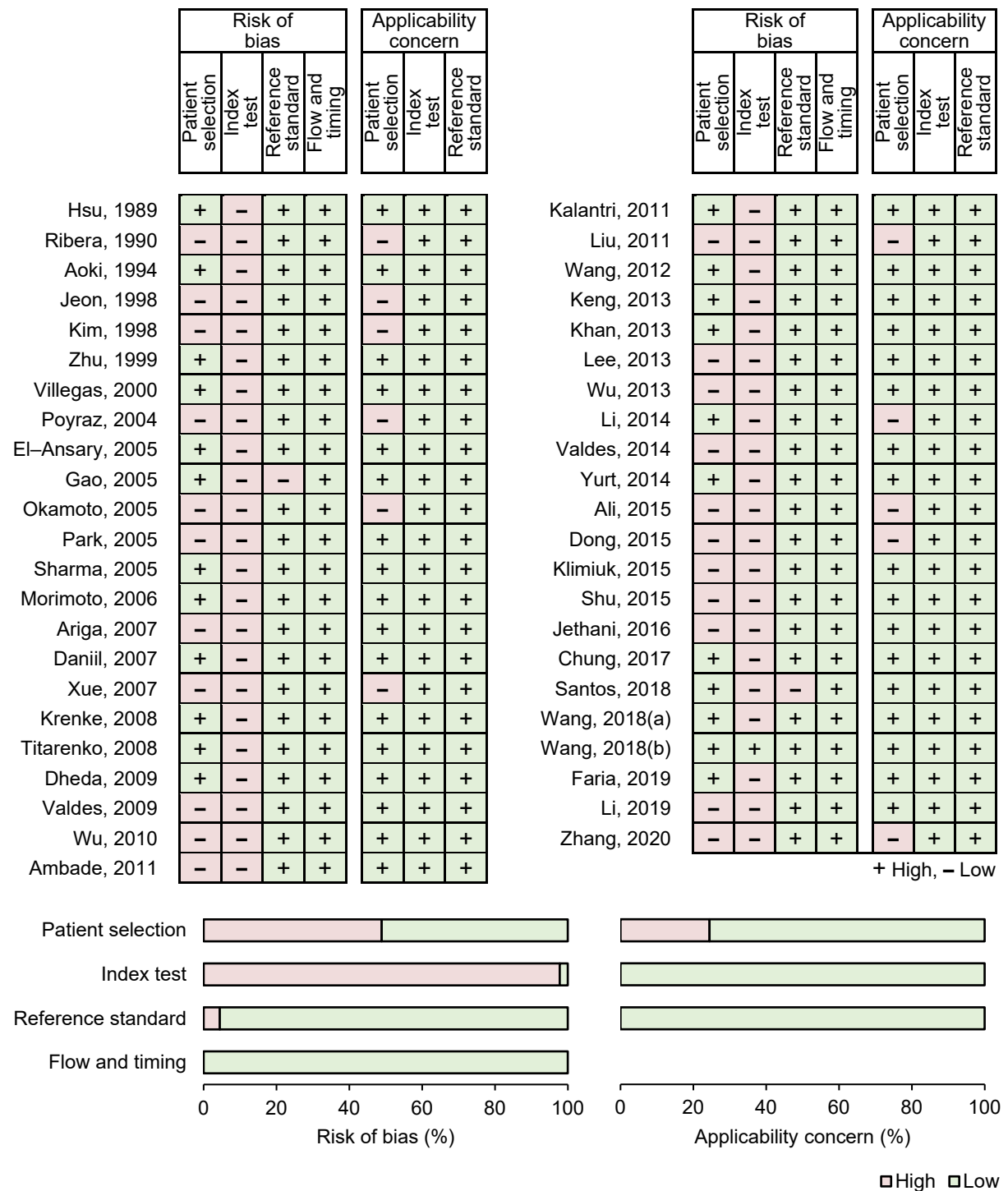

Supplement: S1 Fig — (PDF) [file pone.0253525.s006.pdf]
