# Supplementary material for: Comparative accuracy of pleural fluid unstimulated interferon-gamma and adenosine deaminase for diagnosing pleural tuberculosis: A systematic review and meta-analysis
Source: PLoS One. 2021 Jun 24;16(6):e0253525. doi: 10.1371/journal.pone.0253525 (PMC8224977; doi:10.1371/journal.pone.0253525)

S2 Fig. Deek's funnel plot assessment test for assessing any potential publication bias among studies evaluating pleural fluid adenosine deaminase (left panel) and unstimulated interferon-gamma (right panel). Both these plots show a symmetric distribution of the log of diagnostic odds ratios against inverse root of effective sample sizes, indicating absence of any significant publication bias for either assay.

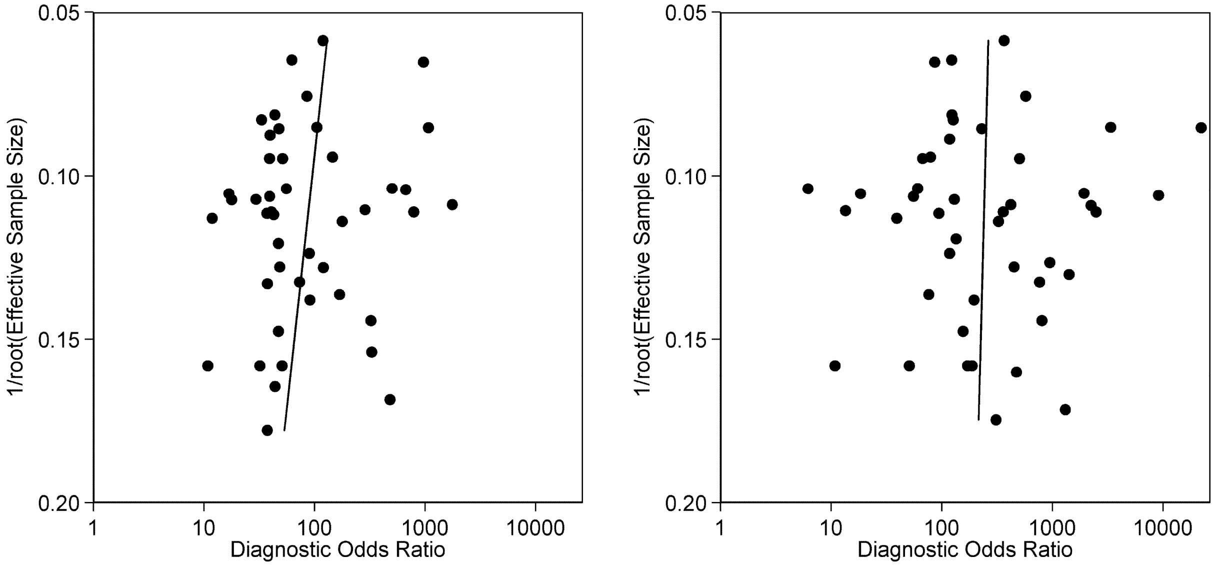

Supplement: S2 Fig — (PDF) [file pone.0253525.s007.pdf]
