# Supplementary material for: Comparative accuracy of pleural fluid unstimulated interferon-gamma and adenosine deaminase for diagnosing pleural tuberculosis: A systematic review and meta-analysis
Source: PLoS One. 2021 Jun 24;16(6):e0253525. doi: 10.1371/journal.pone.0253525 (PMC8224977; doi:10.1371/journal.pone.0253525)

S3 Fig. Coupled forest plot from studies on diagnostic accuracy of pleural fluid adenosine deaminase (ADA) and interferon-gamma (IFN-γ) in the same patient population. Individual sensitivity and specificity estimates are derived from data on true positives (TP), false negatives (FN), true negatives (TN), and false positives (FP), and are represented by solid and hollow squares for IFN-γ and ADA respectively. Horizontal lines depict 95% confidence interval.

Study	Test	TP	FN	TN	FP	Sensitivity	Specificity
Hsu, 1989	IFN-γ	18	1	26	3	0.95 (0.74-1.00)	0.90 (0.73-0.98)
	ADA	18	1	21	8	0.95 (0.74-1.00)	0.72 (0.53-0.87)
Ribera, 1990	IFN-γ	48	0	114	0	1.00 (0.93-1.00)	1.00 (0.97-1.00)
	ADA	48	0	105	9	1.00 (0.93-1.00)	0.92 (0.86-0.96)
Aoki, 1994	IFN-γ	11	0	28	0	1.00 (0.72-1.00)	1.00 (0.88-1.00)
	ADA	9	2	25	3	0.82 (0.48-0.98)	0.89 (0.72-0.98)
Jeon, 1998	IFN-γ	18	2	17	3	0.90 (0.68-0.99)	0.85 (0.62-0.97)
	ADA	17	3	18	2	0.85 (0.62-0.97)	0.90 (0.68-0.99)
Kim, 1998	IFN-γ	39	1	42	0	0.98 (0.87-1.00)	1.00 (0.92-1.00)
	ADA	35	5	41	1	0.88 (0.73-0.96)	0.98 (0.87-1.00)
Zhu, 1999	IFN-γ	24	1	32	1	0.96 (0.80-1.00)	0.97 (0.84-1.00)
	ADA	22	3	30	3	0.88 (0.69-0.97)	0.91 (0.76-0.98)
Villegas, 2000	IFN-γ	45	13	68	2	0.78 (0.65-0.87)	0.97 (0.90-1.00)
	ADA	53	8	60	10	0.87 (0.76-0.94)	0.86 (0.75-0.93)
Poyraz, 2004	IFN-γ	13	2	29	1	0.87 (0.60-0.98)	0.97 (0.83-1.00)
	ADA	13	2	30	0	0.87 (0.60-0.98)	1.00 (0.88-1.00)
El-Ansary, 2005	IFN-γ	14	1	24	0	0.93 (0.68-1.00)	1.00 (0.86-1.00)
	ADA	12	3	22	2	0.80 (0.52-0.96)	0.92 (0.73-0.99)
Gao, 2005	IFN-γ	119	22	47	2	0.84 (0.77-0.90)	0.96 (0.86-1.00)
	ADA	116	25	43	6	0.82 (0.75-0.88)	0.88 (0.75-0.95)
Okamoto, 2005	IFN-γ	10	1	31	1	0.91 (0.59-1.00)	0.97 (0.84-1.00)
	ADA	11	0	31	1	1.00 (0.72-1.00)	0.97 (0.84-1.00)
Park, 2005	IFN-γ	38	1	25	1	0.97 (0.87-1.00)	0.96 (0.80-1.00)
	ADA	38	1	19	6	0.97 (0.87-1.00)	0.76 (0.55-0.91)
Sharma, 2005	IFN-γ	34	1	17	0	0.97 (0.85-1.00)	1.00 (0.80-1.00)
	ADA	32	3	17	0	0.91 (0.77-0.98)	1.00 (0.80-1.00)
Morimoto, 2006	IFN-γ	16	3	43	3	0.84 (0.60-0.97)	0.93 (0.82-0.99)
	ADA	15	4	45	1	0.79 (0.54-0.94)	0.98 (0.88-1.00)
Ariga, 2007	IFN-γ	24	4	45	2	0.86 (0.67-0.96)	0.96 (0.85-0.99)
	ADA	22	5	43	4	0.81 (0.62-0.94)	0.91 (0.80-0.98)
Daniil, 2007	IFN-γ	9	3	47	13	0.75 (0.43-0.95)	0.78 (0.66-0.88)
	ADA	9	3	47	13	0.75 (0.43-0.95)	0.78 (0.66-0.88)
Xue, 2007	IFN-γ	39	6	40	2	0.87 (0.73-0.95)	0.95 (0.84-0.99)
	ADA	36	9	37	5	0.80 (0.65-0.90)	0.88 (0.74-0.96)
Krenke, 2008	IFN-γ	28	0	65	1	1.00 (0.88-1.00)	0.98 (0.92-1.00)
	ADA	28	0	62	4	1.00 (0.88-1.00)	0.94 (0.85-0.98)
Titarenko, 2008	IFN-γ	33	2	51	2	0.94 (0.81-0.99)	0.96 (0.87-1.00)
	ADA	34	1	52	1	0.97 (0.85-1.00)	0.98 (0.90-1.00)
Dheda, 2009	IFN-γ	54	1	19	0	0.98 (0.90-1.00)	1.00 (0.82-1.00)
	ADA	52	3	13	6	0.95 (0.85-0.99)	0.68 (0.43-0.87)
Valdes, 2009	IFN-γ	32	7	53	4	0.82 (0.66-0.92)	0.93 (0.83-0.98)
	ADA	38	1	53	4	0.97 (0.87-1.00)	0.93 (0.83-0.98)
Wu, 2010	IFN-γ	20	3	53	3	0.87 (0.66-0.97)	0.95 (0.85-0.99)
	ADA	22	1	45	11	0.96 (0.78-1.00)	0.80 (0.68-0.90)
Ambade, 2011	IFN-γ	42	6	28	5	0.88 (0.75-0.95)	0.85 (0.68-0.95)
	ADA	38	10	25	8	0.79 (0.65-0.90)	0.76 (0.58-0.89)
Kalantri, 2011	IFN-γ	129	25	48	2	0.84 (0.77-0.89)	0.96 (0.86-1.00)
	ADA	122	32	46	4	0.79 (0.72-0.85)	0.92 (0.81-0.98)
Liu, 2011	IFN-γ	22	2	41	1	0.92 (0.73-0.99)	0.98 (0.87-1.00)
	ADA	17	7	40	2	0.71 (0.49-0.87)	0.95 (0.84-0.99)
Wang, 2012	IFN-γ	71	7	39	5	0.91 (0.82-0.96)	0.89 (0.75-0.96)
	ADA	73	5	40	4	0.94 (0.86-0.98)	0.91 (0.78-0.97)
Keng, 2013	IFN-γ	24	7	55	2	0.77 (0.59-0.90)	0.96 (0.88-1.00)
	ADA	26	5	50	7	0.84 (0.66-0.95)	0.88 (0.76-0.95)
Khan, 2013	IFN-γ	72	0	31	0	1.00 (0.95-1.00)	1.00 (0.89-1.00)
	ADA	62	10	23	8	0.86 (0.76-0.93)	0.74 (0.55-0.88)
Lee, 2013	IFN-γ	55	5	157	3	0.92 (0.82-0.97)	0.98 (0.95-1.00)
	ADA	53	7	147	13	0.88 (0.77-0.95)	0.92 (0.87-0.96)
Wu, 2013	IFN-γ	36	4	40	1	0.90 (0.76-0.97)	0.98 (0.87-1.00)
	ADA	35	5	35	6	0.88 (0.73-0.96)	0.85 (0.71-0.94)
Li, 2014	IFN-γ	38	9	35	8	0.81 (0.67-0.91)	0.81 (0.67-0.92)
	ADA	36	11	36	7	0.77 (0.62-0.88)	0.84 (0.69-0.93)
Valdes, 2014	IFN-γ	63	7	327	34	0.90 (0.80-0.96)	0.91 (0.87-0.93)
	ADA	69	1	337	24	0.99 (0.92-1.00)	0.93 (0.90-0.96)
Yurt, 2014	IFN-γ	32	11	34	16	0.74 (0.59-0.86)	0.68 (0.53-0.80)
	ADA	38	5	44	6	0.88 (0.75-0.96)	0.88 (0.76-0.95)
Ali, 2015	IFN-γ	18	2	19	1	0.90 (0.68-0.99)	0.95 (0.75-1.00)
	ADA	17	3	17	3	0.85 (0.62-0.97)	0.85 (0.62-0.97)
Dong, 2015	IFN-γ	59	4	41	9	0.94 (0.85-0.98)	0.82 (0.69-0.91)
	ADA	45	18	47	3	0.71 (0.59-0.82)	0.94 (0.83-0.99)
Klimiuk, 2015	IFN-γ	43	1	157	2	0.98 (0.88-1.00)	0.99 (0.96-1.00)
	ADA	39	5	148	11	0.89 (0.75-0.96)	0.93 (0.88-0.97)
Shu, 2015	IFN-γ	23	12	58	2	0.66 (0.48-0.81)	0.97 (0.88-1.00)
	ADA	14	21	59	1	0.40 (0.24-0.58)	0.98 (0.91-1.00)
Jethani, 2016	IFN-γ	44	1	44	1	0.98 (0.88-1.00)	0.98 (0.88-1.00)
	ADA	40	5	45	0	0.89 (0.76-0.96)	1.00 (0.92-1.00)
Chung, 2017	IFN-γ	100	6	220	10	0.94 (0.88-0.98)	0.96 (0.92-0.98)
	ADA	93	13	217	13	0.88 (0.80-0.93)	0.94 (0.91-0.97)
Santos, 2018	IFN-γ	29	4	45	1	0.88 (0.72-0.97)	0.98 (0.88-1.00)
	ADA	32	1	39	7	0.97 (0.84-1.00)	0.85 (0.71-0.94)
Wang, 2018(a)	IFN-γ	47	4	98	5	0.92 (0.81-0.98)	0.95 (0.89-0.98)
	ADA	45	6	89	14	0.88 (0.76-0.96)	0.86 (0.78-0.92)
Wang, 2018(b)	IFN-γ	41	3	74	2	0.93 (0.81-0.99)	0.97 (0.91-1.00)
	ADA	39	5	66	10	0.89 (0.75-0.96)	0.87 (0.77-0.94)
Faria, 2019	IFN-γ	14	1	98	7	0.93 (0.68-1.00)	0.93 (0.87-0.97)
	ADA	13	2	98	7	0.87 (0.60-0.98)	0.93 (0.87-0.97)
Li, 2019	IFN-γ	27	17	34	4	0.61 (0.46-0.76)	0.89 (0.75-0.97)
	ADA	33	9	35	3	0.79 (0.63-0.90)	0.92 (0.79-0.98)
Zhang, 2020	IFN-γ	99	9	123	11	0.92 (0.85-0.96)	0.92 (0.86-0.96)
	ADA	94	14	121	13	0.87 (0.79-0.93)	0.90 (0.84-0.95)

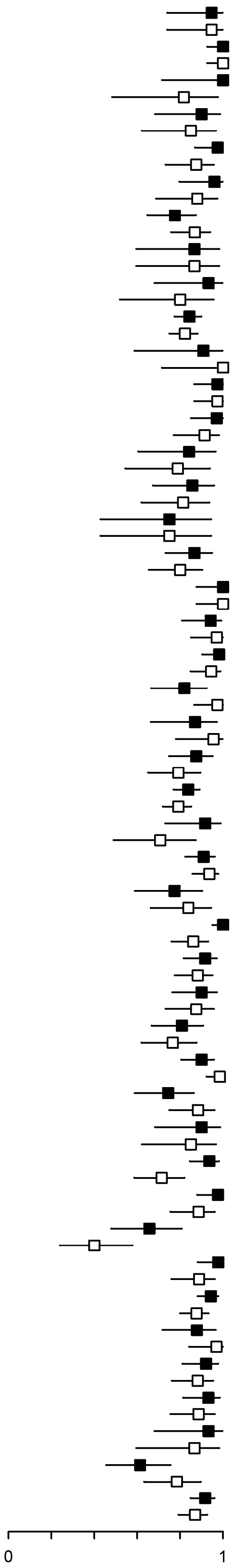

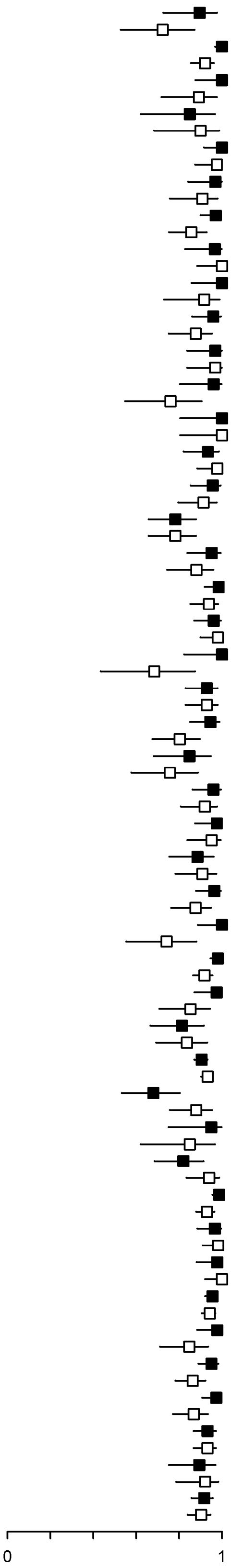

Supplement: S3 Fig — (PDF) [file pone.0253525.s008.pdf]
