# Supplementary material for: Comparative accuracy of pleural fluid unstimulated interferon-gamma and adenosine deaminase for diagnosing pleural tuberculosis: A systematic review and meta-analysis
Source: PLoS One. 2021 Jun 24;16(6):e0253525. doi: 10.1371/journal.pone.0253525 (PMC8224977; doi:10.1371/journal.pone.0253525)

S4 Fig. Likelihood ratio matrix for studies evaluating both pleural fluid adenosine deaminase (red) and interferon-gamma (blue). The hollow circles represent data from individual studies, and the diamond and error lines indicate summary estimates and corresponding 95% confidence intervals of positive and negative likelihood ratios across various studies.

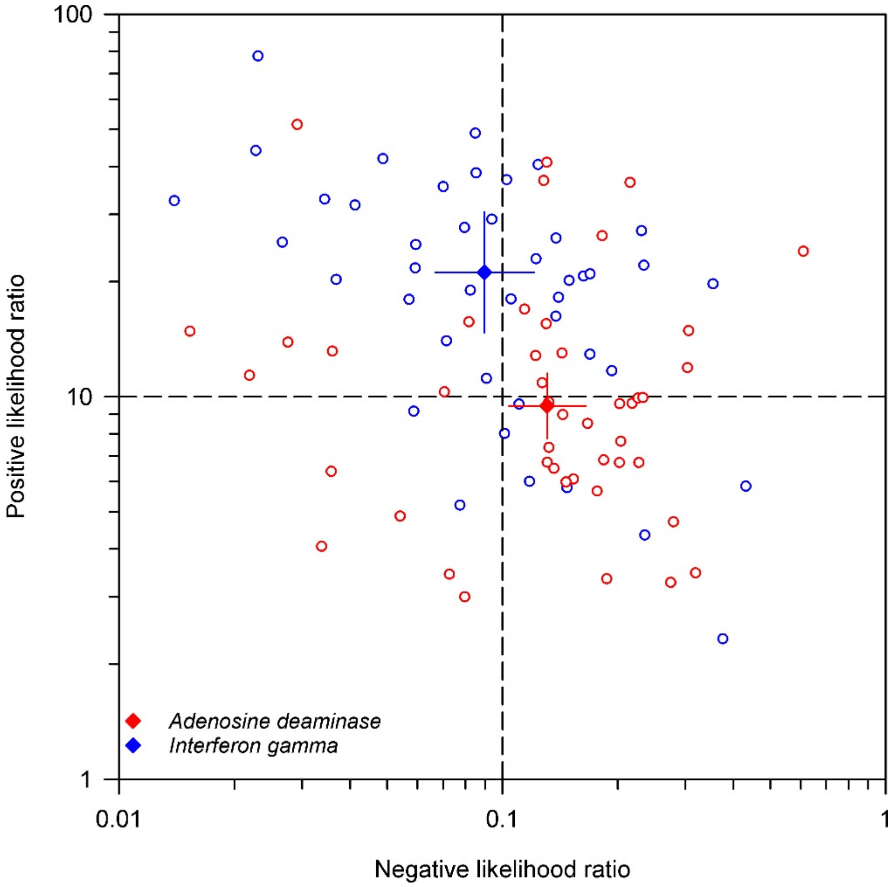

Supplement: S4 Fig — (PDF) [file pone.0253525.s009.pdf]
