# Supplementary material for: Comparative accuracy of pleural fluid unstimulated interferon-gamma and adenosine deaminase for diagnosing pleural tuberculosis: A systematic review and meta-analysis
Source: PLoS One. 2021 Jun 24;16(6):e0253525. doi: 10.1371/journal.pone.0253525 (PMC8224977; doi:10.1371/journal.pone.0253525)

S5 Fig. Summary receiver operating characteristic plots for pleural fluid interferon-gamma (blue) and adenosine deaminase (red) from hierarchical summary receiver operating characteristic (HSROC) modeling. The top left panel shows independent plots for the two index tests. The top right panel shows these plots after adjusting test-type as a covariate and allowing it to influence accuracy, threshold, and shape. The bottom left panel shows these plots after adjusting test-type as a covariate and allowing it to influence accuracy and threshold, but not shape. The bottom right panel shows these plots after adjusting test-type as a covariate and allowing it to influence only accuracy. For each plot, the solid circles indicate the summary diagnostic accuracy points, while the dotted ellipses represent 95% confidence regions around these summary estimates and dashed lines represent the 95% prediction region. See Table E6 for the corresponding model parameter values.

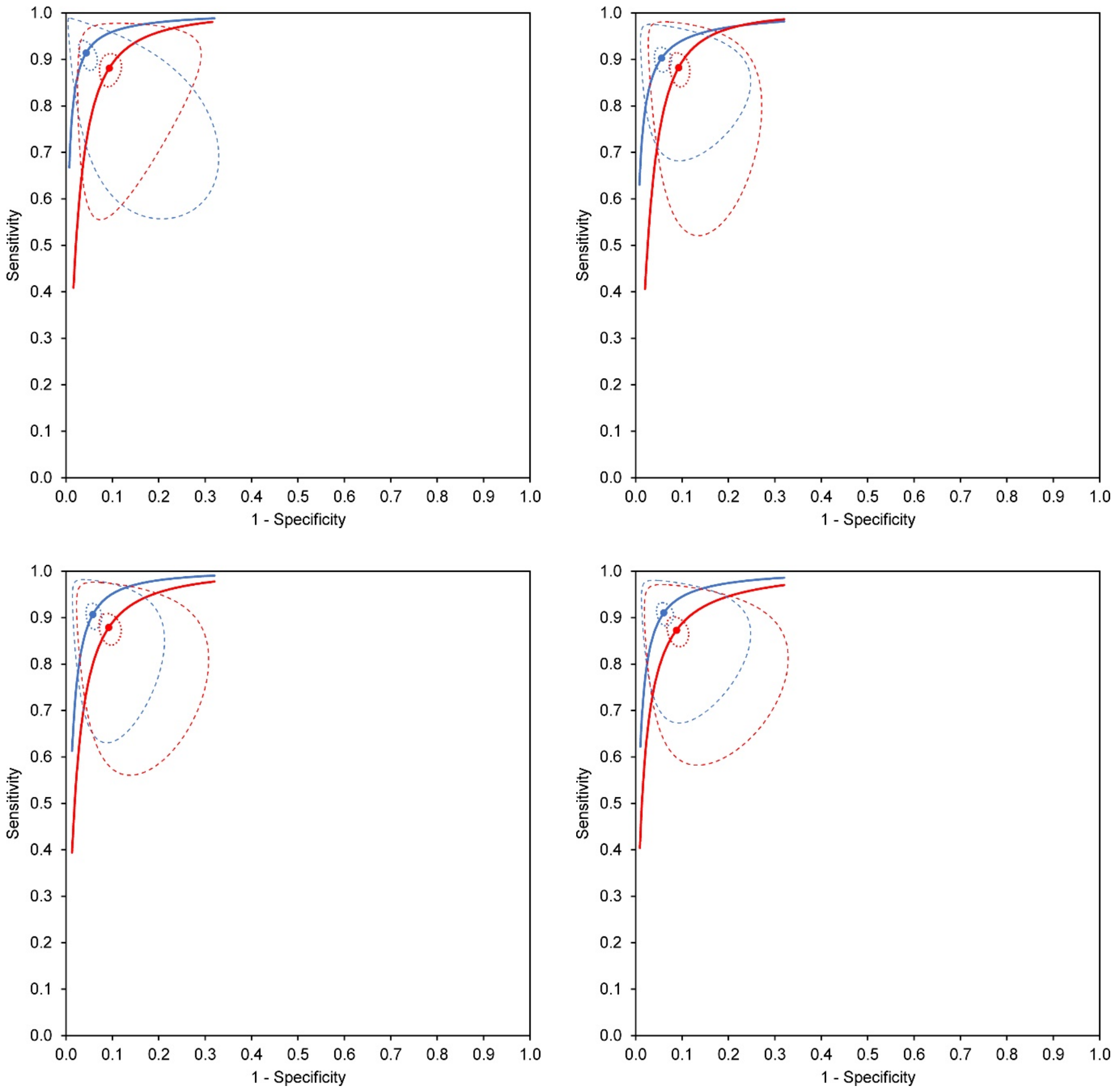

Supplement: S5 Fig — (PDF) [file pone.0253525.s010.pdf]
